# Diversity, Co-occurrence and Implications of Fungal Communities in Wastewater Treatment Plants

**DOI:** 10.1038/s41598-019-50624-z

**Published:** 2019-10-01

**Authors:** Hailemariam Abrha Assress, Ramganesh Selvarajan, Hlengilizwe Nyoni, Khayalethu Ntushelo, Bhekie B. Mamba, Titus A. M. Msagati

**Affiliations:** 1 University of South Africa, College of Science Engineering and Technology, Nanotechnology and Water Sustainability Research Unit, UNISA Science Campus, Florida 1709 Johannesburg, South Africa; 2 University of South Africa, College of Agriculture and Environmental sciences, UNISA Science Florida, 1709 Johannesburg, South Africa; 3 State Key Laboratory of Seperation and Membranes, Membrane Processes, National Center for International Joint Research on Membrane Science and Technologya, Tianjing, 300387 People’s Republic of China

**Keywords:** Water microbiology, Biodiversity, Microbial ecology

## Abstract

Three wastewater treatment plants (WWTPs) located in Gauteng province in South Africa were investigated to determine the diversity, co-occurrence and implications of their fungal communities using illumina sequencing platform and network analysis. Phylogenetic taxonomy revealed that members of the fungal communities were assigned to 6 phyla and 361 genera. *Basidiomycota* and *Ascomycota* were the most abundant phyla, dominated by the genera *Naumovozyma, Pseudotomentella, Derxomyces, Ophiocordyceps, Pulchromyces* and *Paecilomyces*. Phylogenetic analysis revealed the existence of fungal OTUs related to class lineages such as *Agaricomycetes, Eurotiomycetes and Sordariomycetes* indicating new fungal diversity in WWTPs. Dominant and rare fungal genera that can potentially be used in bioremediation such as *Trichoderma, Acremonium, Talaromyces, Paecilomyces, cladophialophora* and *Saccharomyces* were detected. Conversely, genera whose members are known to be pathogenic to human and plant such as *Olpidium*, *Paecilomyces, Aspergillus, Rhodotorula, Penicillium, Candida, Synchytrium, Phyllosticta* and *Mucor* were also detected in all WWTPs. Phylotype analysis confirmed that some fungal phylotypes were highly similar to the reported fungal pathogens of concern. Co-occurrence network analysis revealed that the fungal genera such as *Minimedusa, Glomus, Circinella, Coltricia, Caloplaca, Phylosticta, Peziza, Candida*, and *Hydnobolites* were the major networking hub in the WWTPs. The overall results in this study highlighted that WWTPs represent a potential source of beneficial fungi for bioremediation of pollutants in the ecosystem and the need to consider human and plant fungal pathogens during safety evaluation of treated wastewater for reuse.

## Introduction

Fungi are ubiquitous in the environment where they exist with high diversity, comparable biomass to bacteria and in greater proportion than archaea and viruses^1,2^. Despite their physiological and metabolic differences, they coexist and interact with other group of microorganisms in a variety of environments controlling wide variety of ecosystem functions such as organic decomposition^3^. Particularly, they form important group of microbial communities in wastewater treatment plants (WWTPs)^4^ and aid in degrading environmental organic chemicals, from proteins to complex carbohydrates, lipids, aromatic hydrocarbons, pharmaceutical compounds, heavy metals, endocrine disrupting chemicals by means of wide array of intra-and extracellular enzymes as well as sorption process^5–7^. In addition, they have been associated with denitrification^8^ and stabilization of activated sludge cell aggregates^9^, while their increased biomass is linked to operational problems such as membrane biofouling, foaming and bulking^10,11^.

On the other hand, some pathogenic fungi in WWTPs can be a serious threat to human health since effluents from WWTPs are increasingly being considered not only for the more feasible use in irrigation but also to produce quality water for urban and for drinking^12^. This threat associated with the presence of pathogenic fungi in WWTPs is an issue of concern because fungi are not completely removed by conventional WWTPs and they are not included in regulatory frameworks^13^. Furthermore, they produce mycotoxins, which are considered toxic to humans^14^. Pathogenic fungi also have a significant impact on crop and plant life, influencing food security and the ecosystem^15^. More importantly, the emergence of drug resistant and less susceptible pathogenic fungi in the last decades has also become a great concern on the risks caused by them^16^. All these all observations call for thorough and comprehensive investigation and characterization of fungal communities in WWTPs.

Some studies have reported recently the presence of diverse group of fungal communities in WWTPs^9,17,18^. However, those reports either focused on activated sludge samples or employed culture-dependent methods for fungal identification^17,19,20^. Reports on total fungal communities in raw and treated wastewater from WWTPs are currently very limited in the open literature^21^. This limits our knowledge of the source and role of fungal community in WWTPs as well as understanding of the fungal potential health risks associated with reusing treated wastewaters.

Microorganisms are not commonly encountered in isolation, but establish a community networks that regulate the structure and functions of an ecosystem^22^. Microorganisms show positive or negative co-occurrence patterns where interacting microbes compete or complement in substrate decomposition and nutrient recycling. Microorganisms with similar niches and favors the same substrates can also, however, co-occur through the effect of environmental filtering^23^. Network analysis-based methods have proven to be useful tool to study interactions between microbes and have recently been applied to determine the co-occurrence of microbes in complex environments including soils, pollen, and oceans^24–26^. The application of such network analysis might facilitate the discovery of novel fungal communities which can be used for the co-degradation of pollutants as well as understanding co-pathogenesis. However, there are limited reports in the open literature on the co-occurrence network analysis of fungal communities in WWTPs^4^. In this study, we investigated fungal diversity in wastewater samples from three wastewater treatment plants using Illumina sequencing technology. The aim of this study was to determine the biodiversity of fungal communities in raw and treated wastewater; determine the co-occurrence pattern of the wastewater fungal communities and investigate the implications associated with the occurrence of fungi in wastewater treatment plants (WWTPs).

## Results and Discussion

### Physico-chemical characteristics observations

The results of the physicochemical characteristics of wastewater samples from different wastewater treatment plants were summarized in Table 1. The pH of the water samples ranged from slightly acidic (6.64) to moderate alkaline (8.12). Reported pH of wastewaters is dispersed in a wide range and results observed in this study are comparable with those data reported elsewhere in literature^20,27^. The water physico-chemical parameters such as conductivity (COND), salinity (SAL), dissolved organic carbon (DOC), dissolved oxygen (DO), and major anions noticeably varied among the wastewater samples. Greater variation in the COND of the water samples was recorded and ranged from 685.5 to 902 µ S^−1^ cm^−1^ in the influent samples and 506 to 1016 in the effluent samples. COND values of the influent samples observed in this study falls within the range of CONDs reported by Wang *et al*.^28^. However, slightly higher CONDs were reported for effluent wastewater samples in Turkey^27^. The amount of total dissolved solid (TDS) varied from 341 mg L^−1^ to 513.3 mg L^−1^ in the influent and from 253 mg L^−1^ to 508 mg L^−1^ in the effluent wastewater samples. These values are lower than the TDS measurements recorded by Tanyol and Demir, (2016) for domestic wastewater treatments^27^. In comparison to other water samples, PWWTP water samples showed the highest DOC, TDS, chloride (Cl^−^), fluoride (F^−^), and phosphate (PO_4_^3−^) contents both in its influent and effluent water samples. This could be attributed to the hospital and industrial sewages that the plant receives, in addition to domestic sewage, unlike the other wastewater treatment plants. Nutrients such as bromide (Br^−^), nitrite and nitrate were not detected in the majority of the water samples. Statistically, the level of pH, DO, DOC, TDS, SAL, COND, Cl^−^, F^−^, sulfate (SO_4_^2−^) and PO_4_^3−^were significantly different (P < 0.05) in the studied influents of the wastewater treatment plants (Table 1). Furthermore, significant variation (p < 0.05) in the abovementioned parameters, except DO and fluoride (p > 0.05), were also observed in the effluent wastewater samples.

**Table 1:** Physico-chemical characteristics of the wastewaters studied (mean ± SD, *n* = *3*).

Variables	Wastewater samples							p-value* (Influent, effluent)
	Daspoort Influent (DI)	Daspoort Effluent (DE)	Flip Human Influent (FI)	Flip Human Effluent (FE)	Percy Stewart Influent (PI)	Percy Stewart Effluent (PE)	Sterkfontein Hospital Effluent (HE)	
pH	6.64 ± 0.007	7.05 ± 00.028	7.75 ± 0.007	8.12 ± 0.063	7.15 ± 00.007	7.16 ± 0.014	7.16 ± 0.028	(0.000011, 0.00022)
DO (mg L^−1^)	0.435 ± 0.601	3.45 ± 0.368	0.67 ± 0.028	3.04 ± 0.495	0.48 ± 0.403	1.89 ± 0.785	0.93 ± 0.410	(0.0028, 0.144)
Conductivity (COND) (µ S^−1^ cm^−1^)	840.5 ± 2.121	506 ± 0.000	902 ± 5.656	774 ± 2.828	685.5 ± 6.363	1016 ± 7.07	682 ± 0.000	(<0.00001, 0.000003)
Salinity (SAL) (psu)	0.415 ± 0.007	0.245 ± 0.007	0.445 ± 0.007	0.38 ± 0.000	0.335 ± 0.007	0.505 ± 0.007	0.33 ± 0.000	(0.0001, 0.00005)
NH_3_-N	0.045 ± 0.007	0.06 ± 0.000	0.170 ± 0.042	0.13 ± 0.042	ND**	0.35 ± 0.092	ND	(NA, 0.033)
DOC (mg L^−1^)	20.390 ± 0.620	5.59 ± 0.77	55.13 ± 0.77	11.73 ± 0.67	154.93 ± 2.49	18.16 ± 0.95	63.75 ± 1.15	(<0.00001,<0.00001)
TDS (mg L^−1^)	420.5 ± 0.707	253.0 ± 0.00	451.5 ± 3.536	387.5 ± 2.121	513.3 ± 18.668	508.0 ± 4.242	341.0 ± 0.000	(0.00025, <0.00001)
Cl^-^ (mg L^−1^)	28.641 ± 4.75	41.31 ± 0.338	21.538 ± 0.696	19.338 ± 1.018	60.572 ± 0.002	44.084 ± 2.041	30.295 ± 0.212	(0.0003, 0.00061)
F^−^(mg L^−1^)	0.224 ± 0.095	ND	0.131 ± 0.012	0.204 ± 0.007	16.972 ± 0.489	0.210 ± 0.004	4.85 ± 0.029	(<0.00001, 0.914)
Br^−^(mg L^−1^)	0.543 ± 0.081	ND	0.913 ± 0.053	ND	ND	ND	ND	NA***
Nitrite (mg L^−1^)	ND	ND	ND	ND	ND	4.225 ± 0.119	ND	NA
Nitrate (mg L^−1^)	ND	31.656 ± 0.130	ND	ND	ND	ND	ND	NA
Sulfate (mg L^−1^)	6.689 ± 1.006	40.493 ±	26.988 ± 0.103	22.908 ± 1.248	3.506 ± 0.052	58.110 ± 1.407	15.986 ± 0.051	(<0.00001, 0.00015)
Phosphate (mg L^−1^)	5.076 ± 0.590	ND	12.719 ± 0.089	0.714 ± 0.029	23.285 ± 0.036	4.332 ± 0.008	16.096 ± 0.057	(<0.00001, 0.0085)

*(p < 0.05) defined as statistically significant, **ND-not-detected, ***NA-not-applicable.

Observed concentrations for selected dissolved metals measured in the influent and effluent wastewater samples were summarized in Table S1. All measured metals were detected in all wastewater samples. The results indicated that the level of the measured metals was unevenly distributed across the wastewater treatment plants. Wastewater samples from PWWTP showed higher levels of Ca, Cu, Mn, Ni, and Zn in the influent and Ca, Cu, Fe, Mn, Ni, and Zn in the effluent wastewater samples. On the other hand, higher levels of Fe, and Mg in the influent and Mg, and Mo in the effluent wastewater sample from DWWTP were recorded. FWWTP samples revealed highest concentrations for only Co in its both influent and effluent. Statistical test on the detected metals using one-way ANOVA revealed that there is a significant difference (P < 0.05) in the overall level of the measured metals across the three wastewater treatment plants (Table S1). Furthermore, the metal concentrations obtained in this study greatly vary from the reports of previous studies on wastewater treatment plants^29,30^.

**Figure 1:**
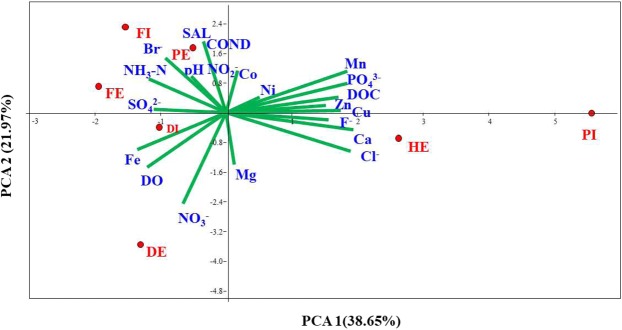
Principal component analysis (PCA) on the physicochemical properties of the wastewater samples from three wastewater treatment plants and a Hospital effluent.

Principal component analysis (PCA) separated the wastewater samples in to three distinct groups (Group 1: PI and HE; Group 2: DI and DE; Group 3: FI, FE, and PE). Figure 1 depicts the distribution of the physicochemical variables formed by the first two axes, which accounted for 62.34% of the total variance. PC1 explained 38.65% of the measured variation, and separated clearly the PI and HE water samples from the rest of the water samples. PI and HE belonging to one group in the PCA might show that HE is the main source or contributor of some of the measured variables. The first component was correlated with DOC, SO_4_^2−^, PO_4_^3−^, Cl^−^, Ca, Zn, Cu and Mn. The second component, accounting for 21.97% of the total variation, was associated primarily with conductivity (COND), salinity (SAL), Br^−^, NH_3_-N, NO_2_^−^, Co, pH and Mg. The overall spatial variation in the concentrations of the measured parameters both in influent and effluent wastewaters of the wastewater treatment plants could be attributed to factors such as the type of raw sewage, capacity of wastewater treatment plant, removal efficiencies, and number of households and industries connected to the wastewater treatment plant.

### Fungal diversity

**Figure 2:**
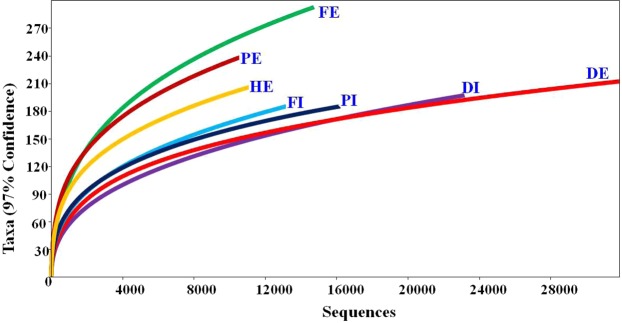
Rarefaction curves describing fungal richness of the analysed samples.

Fungal community exploration in aquatic environment is gaining attention as advanced molecular techniques like next-generation sequencing (NGS) emerge and reveal unexpected abundance, ecological functions, interactions and unclear phylogenetic placements^31^. To date, bacterial communities in wastewater have been widely studied^32,33^. However, studies on fungal biodiversity in WWTPs are still limited, which mostly targeted on activated sludge^18,20^. In this study, culture independent method was employed to determine the fungal biodiversity in both influent and effluent wastewater samples from three WWTPs and one hospital effluent in Gauteng province, South Africa. By using the Illumina sequencing, 10, 561 to 31, 892 effective sequence reads were recorded for each sample, yielding a total of 120,727 quality sequence reads from seven samples. A total of 1,190 OTUs were recorded in the wastewater samples ranging from 145 to 214 OTUs per sample, and accounting for 12 to 18% of the overall OTUs (Table 2). The numbers of quality sequences per sample obtained in this study are mostly lower than sequences reported in a study by Zhang *et al*. (2018) which reported 20,248 to 33,735 effective sequences per activated sludge sample of 18 wastewater treatment plants (WWTPs)^4^. Meanwhile, the number of OTUs recorded in this study falls within the range of OTUs reported by Zhang *et al*.^4^. Furthermore, a study conducted in China determined fungal community in 18 WWTPs and reported 460, 890 quality-reads grouped into 1620 OTUs in total with an average of 8535 sequences and 450 OTUs per sample^20^. The rapid growth at first and turning into a slight plateau of the refraction curve (Fig. 2) showed that the sequencing depth was sufficient to cover most of the fungal diversity and large proportion of fungal community was discovered in each sample. Effluent wastewater samples from Flip-human wastewater (FE) and Daspoort wastewater (DE) treatment plant showed the highest number of OTUs and sequences, respectively (Fig. 2).

**Table 2:** Summary of fungal community diversity indices for samples from three wastewater treatment plants and one hospital effluent.

	Wastewater Sample						
	DI	DE	FI	FE	PI	PE	HE
OTUs	158	166	154	214	145	188	165
Total quality reads	23194	31892	13206	14747	16170	10561	11084
Dominance_D	0.243	0.155	0.096	0.096	0.099	0.060	0.070
Simpson_1-D	0.757	0.845	0.904	0.904	0.901	0.940	0.930
Shannon_H	2.163	2.550	2.959	3.110	2.934	3.529	3.342
Evenness_e^H/S^	0.055	0.077	0.125	0.105	0.129	0.181	0.171
Chao-1	230.1	190.8	239	297.2	168.9	240.1	193.6

Details of the diversity indices calculated to assess within sample complexity of individual fungal population are given in Table 2. The Simpson index (1-D) of all samples revealed few divergences, ranging from 0.757 (DI) to 0.940 (PE). The highest Shannon diversity (H) was observed in PE with 3.52, and the lowest of that was recorded in DI with 2.163 (Table 2). Shannon and Simpson indices found in this study are higher than in the previous report by Gonzalez-Martinez *et al*. (2018), which reported Shannon index ranged from 1.32 to 2.29 and Simpson index (1-D) ranged from 0.54 to 0.81^21^. Shannon index ranging 0.1 to 2.51 and Simpson index (1-D) ranging 0.05 to 0.95 were also reported for fungal community in activated sludge samples^4,20^, which are generally lower than the values obtained in this study. Fungal community in PWWTP samples showed relatively higher Buzas and Gibson’s eveness (e^H/S^) (0.129–0.181), which was about 2.35 times higher than that of DWWTP samples. Comparison of the wastewater samples based on the values of richness measured using Chao1 index showed the highest fungal richness in FE, followed by PE and FI. Jaccard index (Table S2) was used to compare pair wise fungal community similarity between wastewater samples based on the absence and presence of each OTU. The values for the index ranged from 0.34 to 0.47. Effluent samples from Flip Human wastewater treatment plant showed higher dissimilarity with most of the other wastewater samples (Table S2).

### Fungal community structure in wastewater

**Figure 3:**
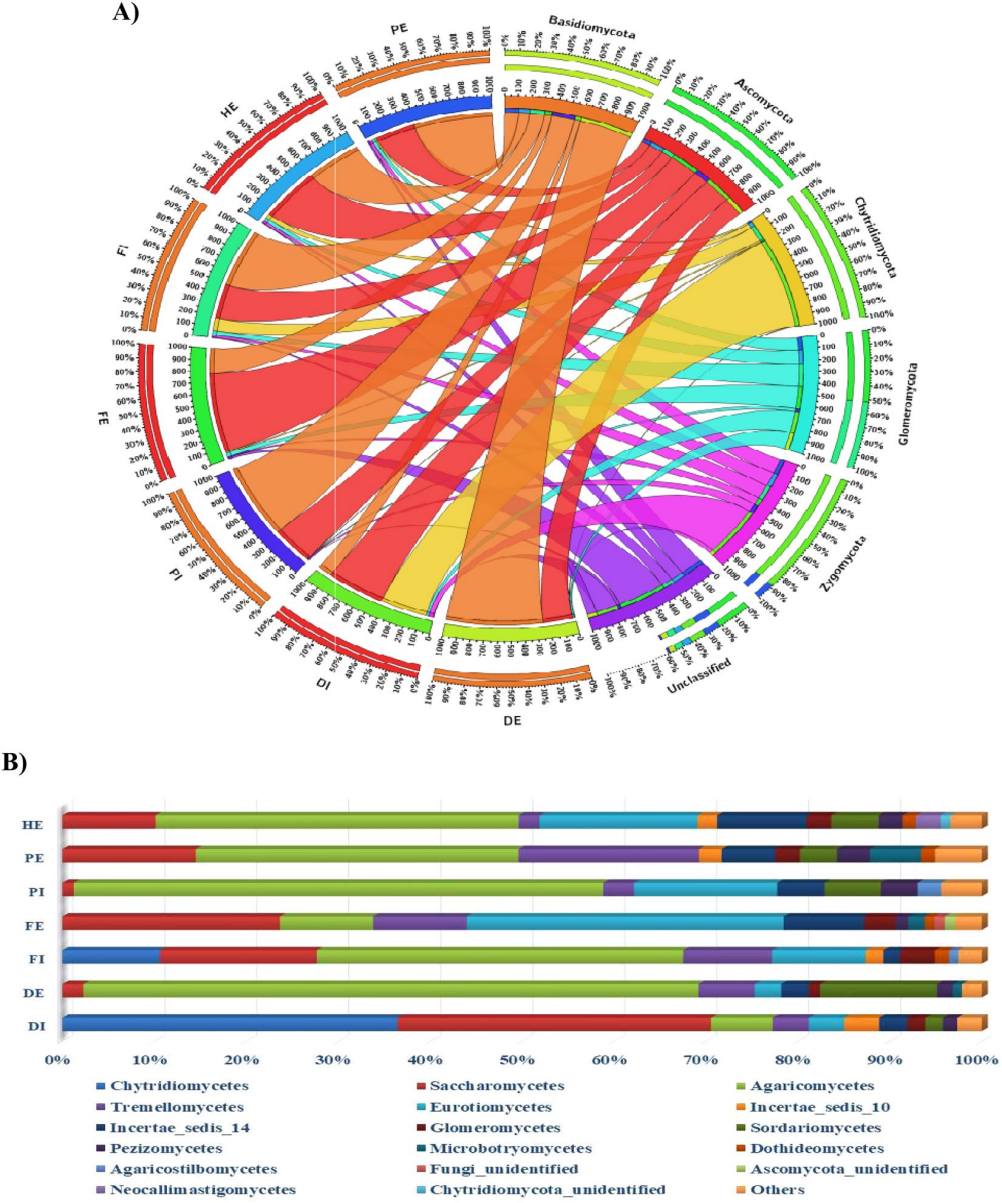
Relative abundance (%) at (**A**) phyla and (**B**) class taxonomic levels of fungal communities in the wastewater treatment plants.

Diverse fungal communities were detected in all the influent and effluent wastewater samples. Overall, 6 fungal phyla together with unclassified fungi, 31 classes, 97 orders, 217 families and 361 genera were observed in wastewater samples collected from three wastewater treatment plants. The fungal abundance observed in the current study were higher than in the previous reported studies of fungal communities in activated sludge from WWTPs^4,20^. Conversely, the number of phyla recorded in this study was lower than those reported in the study by Niu *et al*.^20^. Figure 3 shows the relative abundance at phyla and class taxonomic level (with ≥1% relative abundance) of fungal community in the wastewater samples. The results of taxonomic assignment indicated that *Basidiomycota* and *Ascomycota* were the two most dominant phyla, accounting for 48.38 and 38.36% of the total quality reads, respectively. This was in agreement with previous study of fungal communities in activated sludge from WWTPs^20^, where *Ascomycota* and *Basidiomycota* were also found to be the most dominant phyla. The other phyla accounted 13.16% of the total quality reads, with 0.49% unclassified fungi. *Basidiomycota* accounted for 75, 51.5, 22.28, 64.88, 61.18, and 43.55% and *Ascomycota* accounted for 23.23, 31.40, 70.89, 33.26, 31.29, and 46.93% in wastewater samples from DE, FI, FE, PI, PE and HE, respectively. *Chytridiomycota* (39.4%) and *Ascomycota* (44.99%) dominated in the DI samples. Furthermore, considerable reads corresponding to the phyla *Glomeromycota* (0.41–3.81%) and *Zygomycota* (0.85–3.79%) were also detected in all wastewater samples. The abundance of unclassified fungi ranged from 0.13% in DE to 1.48% in FE (Fig. 3A). Based on the relative abundance results, *Ascomycota* and *Basidiomycota* can be considered as signature phyla for wastewater treatment plants.

Further taxonomic classification at a class level analysis revealed a wider variation in the fungal community of the wastewater different samples (Fig. 3B). *Agaricomycetes* and *Saccharomycetes* were the two predominant classes, constituting 38.93 and 14.26% of the total classes observed, respectively. *Eurotiomycetes, Chytridiomycetes, Tremellomycetes*, and *Sordariomycetes* were the subdominant classes, comprising 10.56, 8.31, 7.04, and 5.38% of the total classes identified, respectively. The classes *Saccharomycetes* (37.12%), *Eurotiomycetes* (27.51%), *Sordariomycetes* (13.99%) and Incertae_sedis_14 (12.30%) mainly represented the phylum *Ascomycota*. Other classes belonging to this phylum such as *Pezizomycetes, Dothiomycetes* and *Leotiomycetes* were also observed at relatively lower abundance. About 0.58% of the classes belonging to the *Ascomycota* phylum were not identified. Among the classes belonging to phylum *Ascomycota*, S*achcaromycetes* was the most abundant class in wastewater samples from DI (75.54%), FI (54.20%), and PE (46.32%). S*ordariomycetes* was dominant in DE (54.77%) while *Eurotiomycetes* in FE (48.46%), PI (46.81%) and HE (36.58%) (Fig. 3B). The class *Agaricomycetes* contributed to about 80% of the total *Basidiomycota* phylum, followed by the classes *Tremellomycetes* (14.53%) and *Microbotryomycetes* (2.81%). Other low abundant classes recorded in the *Basidiomycota* phylum include *Agaricostilbomycetes, Atractiellomycetes, Pucciniomycetes* and *Ustilaginomycetes*. Of the classes related to *Basidiomycota* phylum, *Agaricomycetes* was the most abundant class in all the wastewater samples, except FE, ranging from 57.13% in DI to 90.60% in HE. *Tremellomycetes* was the second most abundant in these samples ranging from 5.16% in PI to 33.32% in DI. *Tremellomycetes* (45.56%) and *Agaricomycetes* (45.29%) were the two most abundant classes in FE wastewater sample (Fig. 3B). The phylum *Chytridiomycota* was represented mainly by c*hytridiomycetes* (92.04%), while the *Glomeromycota* and *Zygomycota* were represented by *Glomeromycetes* (96.32%) and Incertae_sedis_10 (100%), respectively. Classes representing high abundance such as *Agaricomycetes, Tremellomycetes, Microbotryomycetes*, *Sordariomycetes, Saccharomycetes*, *Leotiomycetes*, and *Eurotiomycetes* were reported by previous studies on activated sludge fungal communities from WWTPs^1,2,4,20^. Phylogenetic analysis of the dominant OTUs in the wastewater revealed that the OTUs were divided and related to four main groups (Figure S1). For example, OTUs in clade 1 and 2 were related to the fungal lineage *Incertae sedis* in the phylum *Zygomycota*. Clade 3 and 4 were related to the phylum *Basidomycota* representing new fungal species under the families *Sebacinaceae*, *Albatrellaceae* and *Bankeraceae*. Similarly, OTUs related to the phylum Ascomycota under the classes *Sordariomycetes* (Clade 5) and *Eurotiomycetes* (Clade 6) were also discovered (Figure S1) suggesting that diverse group fungal OTUs in WWTPs needs to be yet identified.

**Figure 4:**
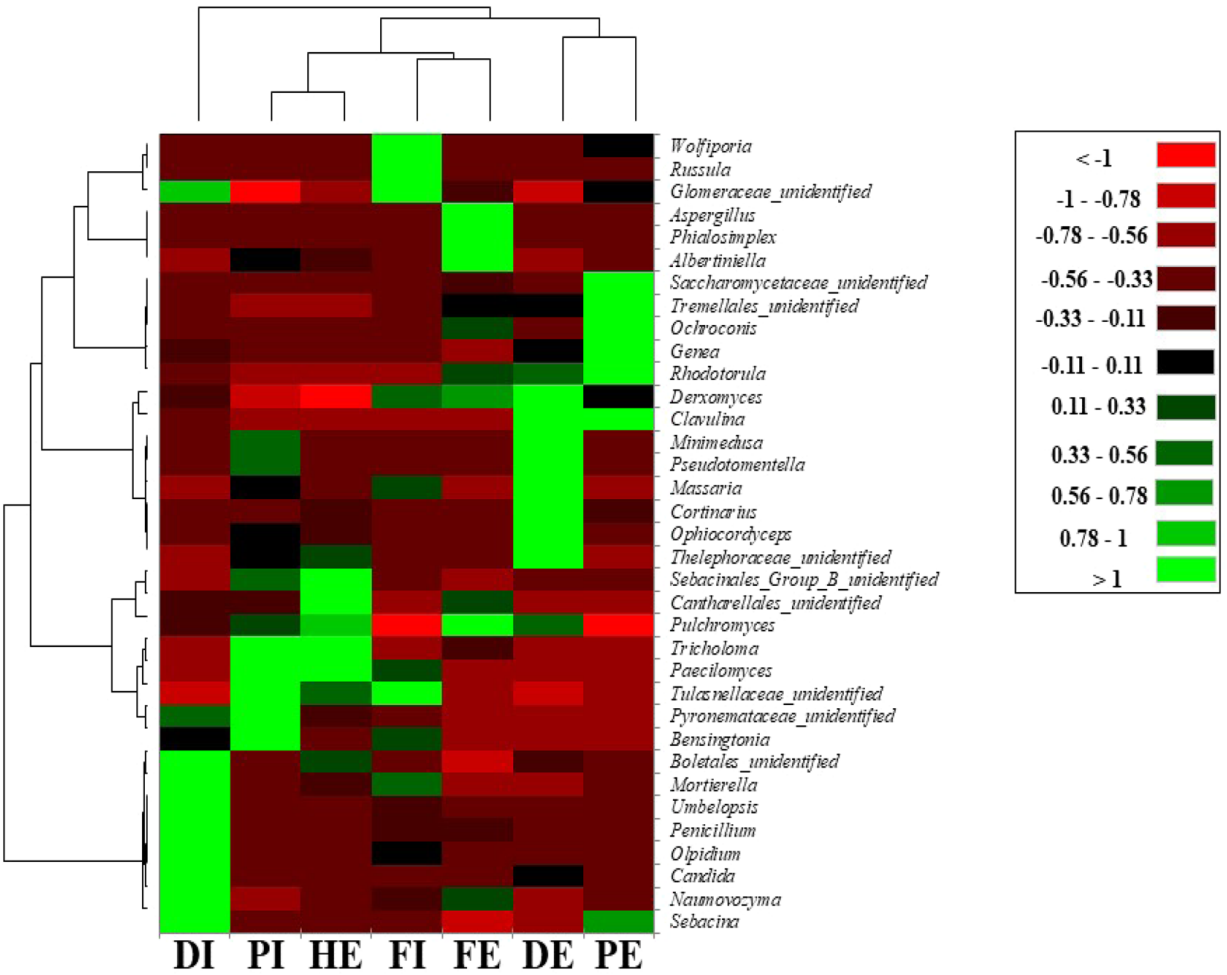
Hierarchically clustered heat map finger printing of the top 10 fungal communities at genus level from each wastewater samples of the wastewater treatment plants and Hospital effluent.

**Figure 5:**
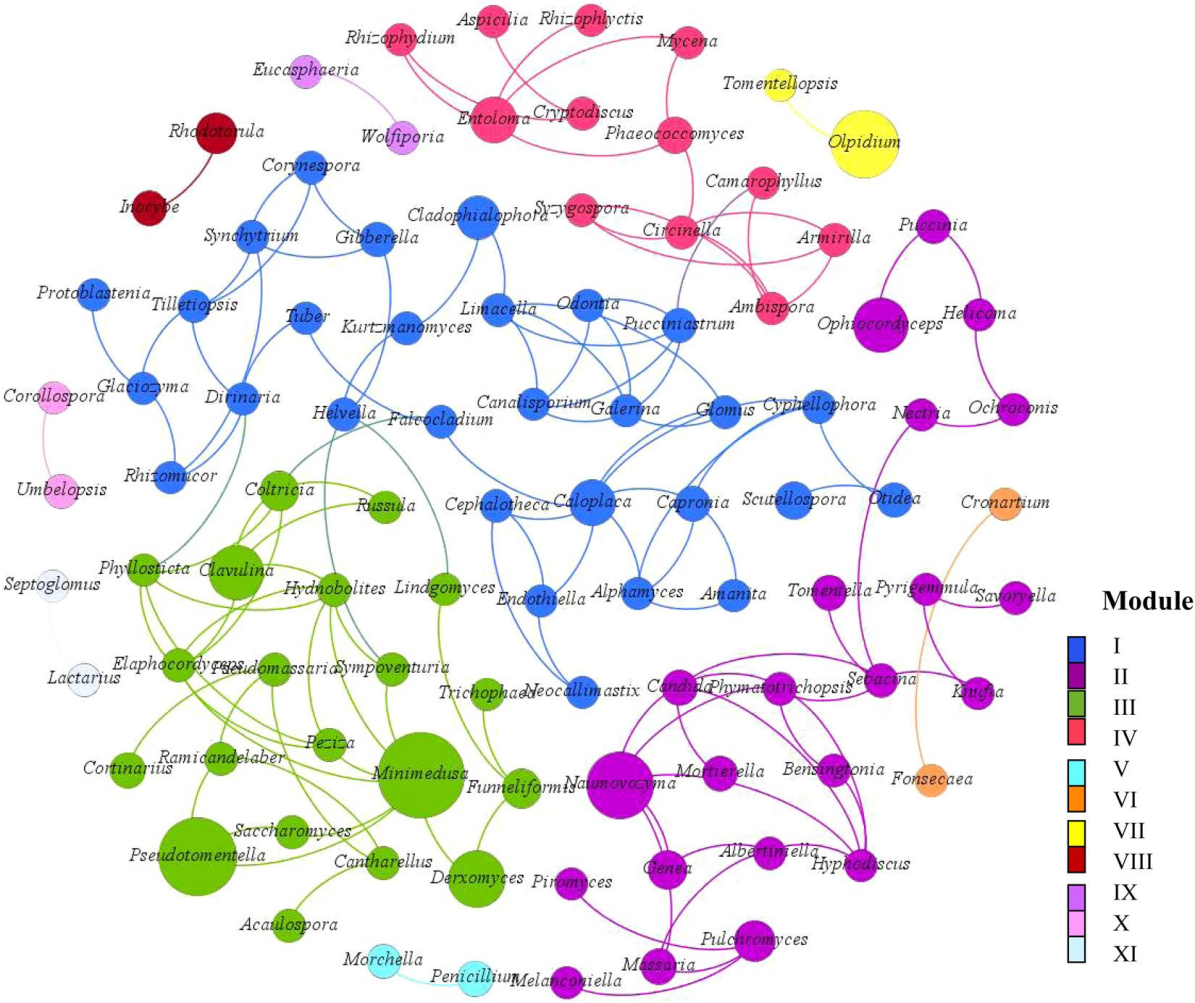
Co-occurrence network of the dominant fungal genera from each wastewater samples. Each connection shows a strong (spearman’s ρ > 0.6) and significant (p < 0.05) correlations. Eleven modules were generated and the same color represents fungal genera with a potential co-occurrence in the same module. The relative abundance of the genera was reflected on the size of each node.

The fungal taxonomies were further classified to a genus level to gain more insight on the community structure. The relative abundance of all 361 genera obtained from all samples was presented in Table S3. Among the 361 detected genera, 75 were shared by all the seven-wastewater samples and accounted for 89.5% of the total count. A total of 214 genera were shared by at least five samples accounting for 95.4% of the total count and from <0.1% to 36% in each sample. 130 unique genera were observed only in one of the samples attributing to about 0.5% of the total count and to <0.05% in each sample. *Naumovozyma* and *Pseudotomentella* were the two predominant genera, comprising 11.91 and 10.59% of the total genera observed, respectively. *Olpidium, Minimedusa, Derxomyces, Ophiocordyceps, Pulchromyces* and *Paecilomyces* were the subdominant genera, constituting 8.22, 7.47, 4.82, 4.72, 4.35, and 4.22% of the total genera identified, respectively. Figure 4 illustrates the profile of fungal community composition in a more detail using a hierarchically clustered heat-map at genus level in order to better assess the different fungal communities in the studied wastewater treatment plants. Ten most abundant genera were selected from each sample (a total of 35 genera of the 7 samples) and used to construct the heat map. As indicated in Fig. 5, remarkable dissimilarities in the fungal composition and relative abundances were observed among the three-wastewater treatment plants. For example, DWWTP (DI and DE) showed higher abundance of *Olpidium, Naumovozyma, Pulchromyces, Derxomyces*, and *Penicillium*, whereas FWWTP (FI and FE), contained higher proportion of *Bensingotonia, Massaria, Derxomyces, Aspergillus, wolfiporia*, and *Russula* (Fig. 4). *Wolfiporia, Tricholoma, Russula, Sebacina, Cortinarius*, and *Albertiniella* were recorded as the most abundant genera in PWWTP. The hospital wastewater samples (HE) was predominated by *Derxomyces, Tricholoma, Cortinarius*, and *Pseudotomentella*. The dominant fungal genera in wastewater revealed here were markedly different from previously reported fungal genera activated sludge. For example, *Niu et al*. (2017) found that *Pluteus, Wickerhamiella, Penicillium*, and *Cryptococcus* were the most abundant genera in activated sludge from 18 WWTPs in china. Other studies on fungal biodiversity in activated sludge from WWTPs in China reported *Guehomyces, Chaetomiaceae, Scutellinia, Geotrichum, Saccharomyces, Malassezia, Debaryomyces, Rhodotorula* and *Candida* as most dominant genera^1,4^. Moreover, Evans and Seviour (2012) examined fungal biodiversity in activated sludge communities based on 18 SrRNA genes using PCR-DGGE and reported *Aspergillus, Cladosporium, Mucor*, and *Penicillium*, as the commonly observed fungal genera^18^. Members such as *Acremonium, Rhodotorula, Candida, Geotrichum, Cladosporium, Sporothrix, Geotrichum candidum, Penicillium, Trichophyton* and *Scopulariopsis* were also reported as frequently occurring fungal genera in activated sludge, aerobic granulated sludge (AGS) and wastewater from WWTPs detected using culture-dependent and independent methods^17,19,34^.

The diversity data presented here showed that influent and effluent wastewater systems harbor specific and diverse fungal communities. Although the three studied WWTPs and the hospital effluent shared several taxonomic groups at all levels, overall composition and diversity of their fungal community appear to differ remarkably. The shared fungal OTUs had higher relative abundance than the rare OTUs, which were largely dispersed and unique to the different wastewater samples. The presence of shared OTUs suggests that there could be core fungal communities in wastewater systems regardless of geographic locations and treatment processes. The unique fungal communities could be attributed to various factors such as the type of sewage, nutrient load, operational parameters, size of wastewater treatment plant and percentage of industrial wastewater received by the WWTPs^4,35^. For example, PWWTP treatment plant receives higher percentage of industrial effluents and hospital effluents than the other wastewater treatment plants (based on oral information).

Direct comparison of the data for biodiversity of fungal communities presented here with other reported information was very difficult because there were no studies reported in the open literature on fungal community in both influent and effluent wastewaters from WWTPs. As a result, the findings of this study were mostly compared with the small number of published papers that reported activated sludge fungal communities in WWTPs. The wastewater samples shared considerable number of fungal communities with the activated sludge at the phyla and class level^1,2,4^. On the other hand, the type and diversity of fungal communities at lower taxonomic levels found in this study deviated from the ones reported previously in activated sludge, i.e., the ones reported as dominant genera in previous studies were either absent or rare in the present study and *vice versa*^1,17,19,34^. To the best of our knowledge, genera such as *Naumovozyma, Pseudotomentella, Olpidium, Minimedusa, Derxomyces, Russula, Massaria, Pulchromyces* and *Paecilomyces* were reported as dominant fungal communities in WWTPs for the first time in this study. The differences in the data between the previous reports and the current study could be attributed to spatial variation, temporal variation, sample type, primers used (18S or ITS), treatment technologies used in the WWTPs and methods used to study the fungal biodiversity (the use of culture-dependent and independent methods)^2,18,19^.

The community of fungi discovered in the studied wastewaters presented strong evidence of the potential of the fungal communities to contribute to the removal of organic pollutants. The data also revealed that wastewaters could be used as a possible source for isolation of different groups of fungi, which can be studied and characterized for the degradation of emerging pollutants in the ecosystem. *Ascomycota* are the largest group in both aquatic and terrestrial fungal kingdom, contributing for greater than 65% of the currently known fungi and key fungal communities for the transformation of pollutants and decomposition of organic matter in nature^2,36^. Many of the dominant and rare fungal genera identified in this study belonging to the class *Sordariomycetes* (*Trichoderma, Fusarium, Acremonium, Cunninghamella*) and Eurotiomycetes (*Aspergillus, Penicillium, Talaromyces, Paecilomyces, Cladophialophora*) are capable of transforming organic contaminants such as toluene, polyaromatic hydrocarbons (PAHs), dioxins, thiocyanate, synthetic dyes, polychlorinated byphenyls, pesticides, and plastics^2,37,38^. Enhanced dewaterability and filterability of activated sludge by members of *Penicillium* during treatment process genera is also reported^39^. Members of the class *Saccharomycetes* (*Pichia, Candida, Saccharomyces*) are known to degrade phenol, hydrocarbons, remove chromium, 2,4,6-trinitrotoluene (TNT), the endocrine disrupting chemical (EDC) nonylphenol, PAHs, *n*-alkylbenzenes, n-alkanes and crude oil^5,40,41^. Furthermore, members of the genera *Fusarium, Gibberella, Nectria, Trichoderma, Hypocrea*, and *Hypomyces* were shown to have denitrification abilities^8^. Members of the phylum *Basidiomycota*, which mostly inhabit terrestrial environments and accounts for about 34% of the known fungal species, are also known to mineralize organic pollutants^8^. For example, *Trichosporon* (class *Tremellomycetes*) and *Rhodotorula* (class *Microbotryomycetes*) are involved in COD removal and metabolism of PAHs, cresols and phenolic compounds^1,5,42^. Subdivisions of fungi Incertae sedis, which belong to the phylum *Zygomycota*, were previously regarded as unable to degrade pollutants (Maza-Márquez *et al*.^2^. However, recent studies showed that this division of fungi includes some well-studied species that are capable of transforming xenobiotics^2,43^. Contrary to their role in treatment, the occurrence of fungi in WWTPs has been also associated with other implications such as foaming, bulking, and membrane fouling. *Trichosporon* and *Candida* are the two genera which are frequently reported in this regard^44,45^.

Apart from their potential role in bioremediation, several fungi occurring in wastewater are also potential human and plant pathogens. Fungal genera previously related with human and plant pathogens were detected in this study (Fig. S2). This implies that these pathogens could be a threat to human and plant health when treated effluent from the studied WWTP is abstracted for crop irrigation as well as used as direct or indirect source of drinking water. Among the potentially pathogenic fungi detected include dominant and rare genera such as *Candida, Aspergillus, Acremonium, Mucor, Talaromyces, Trichosporon, Rhodotorula, Fusarium, Penicillium, Cladosporium, Ochroconis, Puccinia, Ceratocystis, Synchytrium, Olpidium, Phyllosticta*, *Sporothrix, Cladophialophora, fonsecaea, Lacazia, Phymatotrichopsis, Mycosphaerella, Ustilago*, and *Paecilomyces*^46–50^. *Olpidium*, *Paecilomyces, Aspergillus, Rhodotorula, Penicillium, Candida, Phymatotrichopsis, Ochroconis, puccinia, cladophialophora, Synchytrium, Phyllosticta* and *Mucor* were detected in all WWTPs (Fig. S2). *Olpidium* was the most abundant pathogenic genera accounting 8.22% of the total genera recorded in this study, followed *by Paecilomyces (*4.22%*), Aspergillus (*1.95%) and *Rhodotorula* (1.33%). *Penicillium (0.63%)* and *Candida (0.37%)* were the subdominant pathogenic fungi. *Cladosporium, Acremonium, Ceratocystis, Mycosphaerella, Ustilago* and *Lacazia* were rare fungal genera detected only in one WWTP, having a low relative abundance.

Above 90% of all reported human deaths related to fungal infections are caused by species that belong to either *Aspergillus, Cryptococcus, Pneumocystis* or *Candida*^15^, of which *Cryptococcus* and *Pneumocystis* were not detected on this study. FWWTP showed the highest count of *Aspergillus* genera, accounting about 4% of the total genera observed. Further, phylotype analysis confirmed that OTUs belonging to these genera had closest similarities to the pathogenic fungal species including *Aspergillus fumigatus, Aspergillus niger* and *Aspergillus flavus* (Table S4). Exposure of immunocompromised patients to *Aspergillus* sp. translates into common and life-threatening infections such as chronic pulmonary aspergillosis, pulmonary and nasal allergies, asthma, pneumonitis and hypersensitivity^15,51,52^. *Candida* species are the common pathogens for oropharyngeal, esophageal, cutaneous, invasive, and vaginal candidiasis^53,54^. The highest count (about 80%) of *Candida* genera was recorded in DWWTP.

Fungal infections caused by species other than *Candida* and *Aspergillus* species are also becoming a growing concern due to the increase in the number of infections^55^. For instance, *Rhodotorula* species are presented as causative agents of meningitis, fungemia, peritonitis, endophathalmitis, skin, and prosthetic infections in immune paired patients^55,56^. Some *Penicillium* species are involved in superficial, invasive infections and allergies^57^. The genus *Trichosporon*, which have 17 known species, is found to contain the same risk factors for fungemia as *Candida* species^55^. Some species of the rare genus *Cladosporium* is linked with allergic rhinitis, respiratory arrest in asthmatic patients, and phaeohyphomycosis^58^. *Paecilomyces* species are associated with the infection of eye, skin, soft tissue, heart and lung during immunosupression, surgery, foreign body plant, and trauma^59^. In this study, fungal sequences exhibiting high identity scores with GenBank and belonging to the genera *Penicillium* and *Cladosporium* were identified. Fungal sequence phylotypes OTU56 and OTU28 showed 97–99% sequence similarity with the pathogenic species *Penicillium crustosum and Cladosporium sphaerospermum*, respectivel*y* (Table S4).

Lastly, it is estimated that fungal infections cause about 10% lost in agricultural production every year^60^. For instance, species of the fungal plant pathogen *Synchytrium* causes potato wart^61^, while some species of *Phyllosticta* and *Penicillium* are related with Citrus Black spot disease (CBS) and postharvest citrus infection, respectively^62,63^. It has also been reported that the filamentous *Ascomycetes* of genera *Fusarium, Aspergillus*, and *Penicillium* are responsible for mycotoxins contamination of crops such as maize and groundnut^64^. OTUs with high identity similarity to species of concern in plant fungal pathogen such as *Olpidium bornovanus, Phymatotrichopsis omnivora* and *Synchytrium endobioticum* were shown in Table S4. Generally, while high similarity of fungal phylotypes cannot confirm that they represent the same biological species^65^, our results suggests that WWTPs can be hot spots for potential human and plant pathogens which can pose a series threat on human particularly, and the ecosystem as a whole. Adequate treatment before release, monitoring and evaluation of the effluent as well as inclusion of the persistent fungal pathogens in the regulatory limits are therefore recommended to minimize the harmful effects of fungal pathogens existing in WWTPs.

### Fungal co-occurrence network analysis

The fungal co-occurrence patterns were assessed in this study using network inferences based on strong spearman’s rank analysis (ρ > 0.6; P < 0.05) and the genera with relative abundance greater or equal to 0.05% in each sample. Hundred and ten dominant genera were used for the co-occurrence network analysis after excluding the relatively low abundant genera and other unclassified genera. The resulting network consisted of 97 nodes and 131 edges, where nodes represent fungal genera and edges significant positive spearman’s correlation among genera. The network had 11 modules of closely associated genera, each indicated by color (Fig. 5). The network was characterized by average degree of 2.71, network diameter of 18, modularity index of 0.707, average clustering coefficient of 0.352 and average path length of 6.03. Modules I, II, III, and IV showed higher number of nodes having 29, 22, 20, and 12 nodes, respectively. Based on the network connectivity statistics such as degree centrality, closeness centrality and betweenness centrality, fungal genera including the *Minimedusa, Glomus, Circinella, Coltricia, Caloplaca, Phylosticta, Peziza, Candida*, and *Hydnobolites* were among the hubs that served as the main connecting nodes.

*Basidiomycota* and *Ascomycota* were the most dominant phyla in the co-occurrence network, which indicates that these phyla are capable of adapting in different environments. The fact that some fungal genera such as *Minimedusa, Glomus, Candida, Peziza*, and *Hydnobolites* had more connections could indicate the importance of these genera in the co-occurrence network. For example, some species of *Minimedusa* that utilizes polysaccharides, hexose and oligosaccharides, are known to translocate nutrients and concentrate biogenic microelements (N, P, S, K and Ca)^66^. The human pathogen *Candida* is related with formation of biofilms through surface adhesion and extracellular polymers^45^, which is known to play a key role in the exclusion of nutrient competition and environmental stress^67^. Some species of *Glomus* are reported to drive the microbial diversity and function in their sphere by releasing polysaccharides and water-soluble sugars *via* their hyphae^68^. On the other hand, it could also mean that these hubs are primary organisms involved in the breakdown of organic pollutants in the wastewater^4^. In general, establishing whether the co-occurrences observed in this study are the result of specific biotic interaction, co-operative metabolism or environmental filtering was difficult due to the scarcity of data on the physiology of the interacting partners. Therefore, future researches should focus on understanding the physiology of the hubs and types of interactions within the co-occurrence network. Understanding the interactions in the network could help in exploring novel bioremediation strategies and management of co-pathogenesis.

### Influences of physico-chemical variables on the fungal community

**Figure 6:**
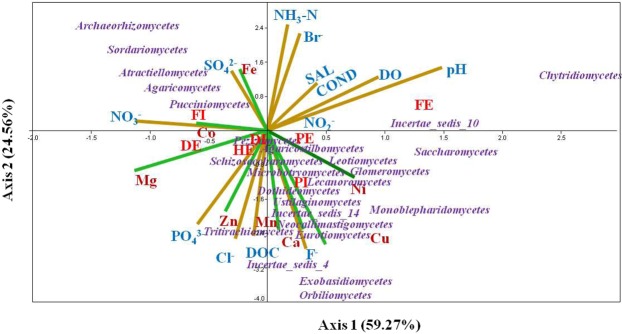
Canonical correspondence analysis (CCA) of the relation between fungal community compositions and physicochemical properties of wastewater samples from three wastewater treatment plants and a hospital effluent.

Ecological studies widely use multivariate analysis to explain the effect of physicochemical parameters on abundance of microorganisms. In this study, canonical correspondence analysis (CCA) approach was used to determine possible relationship between the measured physicochemical variables and fungal communities (at class level) in wastewater treatment plants (Fig. 6). In this analysis, axis-1 and axis-2 explained 84% of the correlation between the wastewater samples, physicochemical properties and the fungal communities (Fig. 6). CCA bi-plots indicated that wastewater physicochemical variables remarkably influenced fungal communities. For instance, the distribution of the classes such as *Pezizomycetes, Lecanoromycetes, Agaricostilbomycetes, Schizosaccharomycetes* and *Dothideomycetes* were influenced by combination of factors including PO_4_^3−^, Cl^−^, DOC, NO_3_^−^, Mg, and Zn concentrations. While, the members belonging to the classes such as *Eurotiomycetes, Exobasidiomycetes, orbiliomycetes, Glomeromycetes, Saccharomycetes* and *Leotiomycetes* were correlated to DOC, F^−^, Cl^−^, PO_4_^3−^, Ca, Ni and Mn levels. NO_3_^−^, SO_4_^2−^, Co, and Fe were the main environmental parameters influencing the fungal community belonging to the classes *Agaricomycetes, Pucchinomycetes, Atractiellomycetes, Sordariomycetes*, and *Archaeorhizomycetes*. On the other hand, pH, DO, Br^−^, COND and SAL showed no correlation with the majority of the fungal classes detected. The findings in this study are in agreement with previous study in which the influent characteristics were reported to significantly influence the abundance of yeasts/*Saccharomycetes*/ in activated sludge from WWTPs^35^. Elsewhere, pH, salinity, temperature, DOC, particulate organic carbon (POC), total organic carbon (TOC), total nitrogen (TN), electrical conductivity, NH_4_^+^-N, NO_3_^-^-N, total phosphorus (TP), DO, and biochemical oxygen demand (BOD) were described as factors driving microbial community structure in different habitants^4,28,69–71^. There is little information in the open literature on the effect of level of metals on diversity and community structure of fungi. Moreover, the available reports are only limited to few metals and inconsistent to each other^72^. For example, a study by Op De Beeck, M. *et al*. (2015) on impact of metal pollution on fungal diversity and community structures showed that Zn and Cd contents were strongly correlated with the fungal community composition but not with their diversity^72^. On the other hand, change community composition and increase in abundance of soil fungi due to increased Zn concentration was reported in a study done in New Zealand^73^.

## Conclusions

The biodiversity and structure of fungal community in both influent and effluent wastewater samples from three different WWTPs were revealed based on the high-throughput illumina sequencing. The results showed that the WWTP fungal communities were grouped into 6 phyla, 31 classes, and 361 genera*. Basidiomycota* and *Ascomycota* were the two dominant phyla encountered across all WWTPs. *Agaricomycetes* and S*accharomycetes* were the two predominant classes, while *Naumovozyma* and *Pseudotomentella* were the two predominant genera. PCA results showed an overall spatial variation in the physicochemical characteristics of the wastewater samples. CCA demonstrated that physicochemical properties such as DOC, PO_4_^3−^, pH, Nitrate and nutrients such as Zn, Ca, Cu, Mn, and Mg remarkably affected the fungal community. Fungal genera which are previously reported as relevant genera for the biodegradation of trace organic pollutants and organic matter, as well as fungal genera that are known to be involved in WWTP operational issues such as membrane biofouling, bulking and foaming were detected. Fungal OTUs that belong to genera of plant and human fungal pathogens were also detected. The data presented in this study implied the role of different fungi that can be played in treatment of wastewater if WWTPs are engineered in consideration of such bio-resources as well as the necessity for including opportunistic fungal pathogens during safety evaluation of the reuse of treated wastewater for different purposes.

## Experimental

### Study area

**Figure 7:**
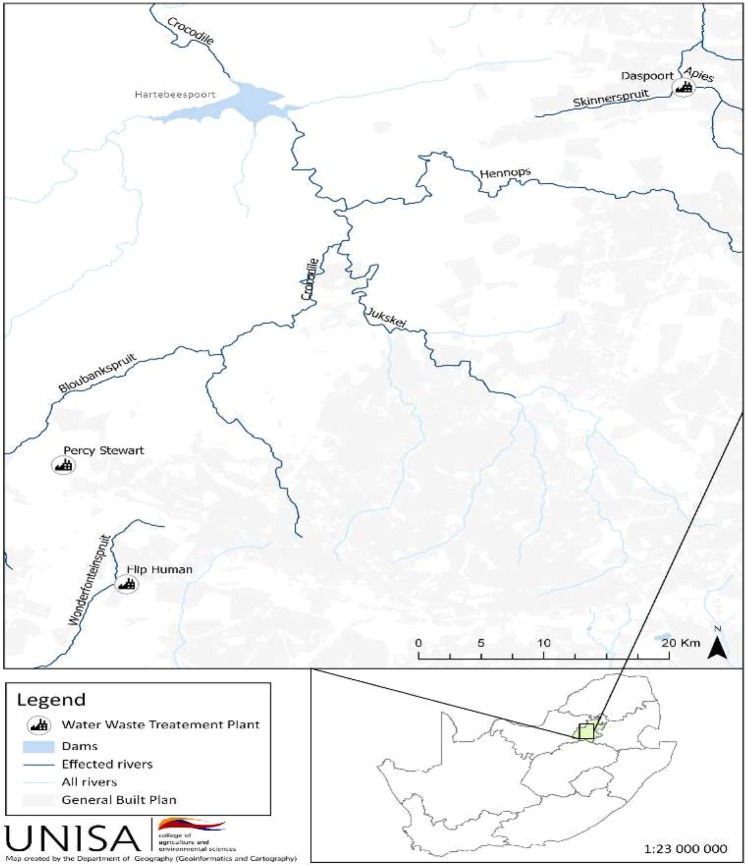
A map showing the sampling points of waste water treatment plants and the effected river streams in Gauteng province, South Africa.

Gauteng is the smallest province in South Africa, accounting for less than 2% of the land area, and the most densely populated as well as urbanised province in the country. To this effect, water samples were collected from three wastewater treatment plants and one hospital effluent in Gauteng province. The three WWTPs included the Daspoort wastewater treatment plant (DWWTP) from Pretoria and Flip Human wastewater treatment plant (FWWTP), Percy Stewart wastewater treatment plant (PWWTP) from Johannesburg (Fig. 7). All the wastewater treatment plants treat the incoming wastewater based on trickling filter and activated sludge technology, but with different treatment capacities.

PWWTP receives its influent from households and industries located in Krugersdorp, Gelita and Millside and the treated effluent from the plant is discharged to the Bloubankspruit river which links up with the Crocodile River and then flows into the Hartbeespoort Dam, which is the main source of irrigation and drinking water for the local community in Hartbeespoort area, Northwest province in South Africa. Hartbeespoort Dam is also used for recreational purposes and is a major tourist attraction hotspot. While, FWWTP and DWWTP receives mostly domestic sewages from surrounding households, following the treatment, FWWTP discharges its treated effluents into the upper Wonderfonteinspruit, which is part of the Mooi River catchment system, which in turn, flows to Vaal Dam. Rand water supplies drinking water to people in Gauteng and surrounding areas from Vaal Dam. The effluent from DWWTP is discharged to Apies River that flows down to the Pienaars River which is one of the tributaries of Crocodile River.

### Sample collection and field measurements

Two liters of wastewater (influent and effluent) were sampled from each WWTP in sterile sample containers. However, the hospital effluent (from Sterkfontein hospital) was collected at PWWTP before it mixed with other sewages from elsewhere. Collected wastewater samples were immediately transported to the laboratory in an icebox for further analysis. One litre of the collected wastewater samples was divided into two equal portions (each 500 mL), one half was stabilized with 5 mL nitric acid for heavy metal analysis while the other half was used for the determination of nutrients and dissolved organic content (DOC). Remaining one-litre samples were used for total DNA extraction. Physico-chemical parameters such as dissolved oxygen (DO), conductivity, salinity, ammonia nitrogen (NH_3_-N), total dissolved solids (TDS) and pH were measured in the field with YSI professional plus (Xylem Inc., USA)

### Determination of anions using Ion chromatography

Metrohm ion chromatograph 861 (Herisau, Switzerland) equipped with conductivity detector (adjusted to full scale of 250 µS cm^−1^) and Metrohm suppressor module (MSM) was used for the chromatographic determination of nutrients (chloride, fluoride, nitrite, bromide, nitrate, phosphate and sulfate). The separation was carried out on a Metrosep A supp 5 (250 × 4 mm) anion exchange column. Briefly, 50 mmol L^−1^ of sulfuric acid (H_2_SO_4_) was used for continuous regeneration of the suppressor. A mixture of 3.2 mmol L^−1^ Na_2_CO_3_ and 1.0 mmol L^−1^ NaHCO_3_ was used as an eluent, while the total run time was 30 minutes. Mullti-anionic standard solution (PerkinElmer, USA) containing the target anions with stock concentration of 100 mg L^−1^ was used to prepare calibration curve in the range of 0.16 to 100 mg L^−1^. Samples were pre-filtered using 0.45 µm acrodisc® syringe filters GHP membranes (PALL life sciences, USA) and injected in triplicates. IC.Net 2.3 (Metrohm) software was used for data acquisition and data analysis.

### Determination of dissolved organic content (DOC)

The dissolved organic carbon (DOC) was determined with total organic carbon (TOC) Analyser equipped with a high-pressure Non-Dispersive Infrared (NDIR) detector and auto sampler (Teledyne Tekmar, Torch model, USA). Prior to the measurement, the samples were filtered with 0.45 µm acrodisc® syringe filters with GHP membranes (PALL life sciences, USA). The DOC content was measured by using a high temperature catalytic oxidation method performed at 700 °C and subtracting the measured inorganic carbon (IC) from the measured total carbon content. Samples were measured in triplicates with an injection volume of 3 mL. A Six-point calibration curve was constructed using standard solutions ranging from 0 to 20 mg L^−1^ of potassium hydrogen phthalate (KHP).

### Determination of selected metals using ICP-MS

Wastewater samples stabilized for ICP-MS were pre-filtered using 0.45 µm acrodisc® syringe filters with GHP membrane (PALL life sciences, USA). A total of eight (Fe, Cu, Mn, Mg, Zn, Co, Ca, and Ni) were then Analysed by directly aspirating the samples in to NeXION 350D Inductively Coupled Plasma Mass spectrometry (ICP-MS) (PerkinElmer, USA). The instrumental operating conditions for the determination of the metals include; Cyclonic spray chamber, 1400 kW RF power, 1.2 L min^−1^ auxiliary gas flow rate, 15.5 L min^−1^ plasma gas flow rate, 6 mL min^−1^ sample uptake and 50 ms dwell time. NeXION set up standard solution containing Al, Mg, U, Ce, CeO and Ti was used for standard performance check. The analytical masses used for analyzing the metals were Fe (55.9349), Ca (43.9555), Co (58.9332), Cu (62.9298), Mg (23.985), Mn (54.9381), Ni (59.9332) and Zn (65.926), atomic mass unit (amu). Five serially diluted standard solutions ranging from 0.1 mg L^−1^ to 1 mg L^−1^ prepared from multi-element standard solution (PerkinElmer, USA) using 1% nitric acid were used to construct a calibration curve for quantification. Syngistix for ICP-MS (PerkinElmer, USA) software was used for data acquisition and data analysis. Samples were Analysed in triplicates.

### DNA extraction, illumina sequencing and data analysis

The influent and effluent samples collected for fungal diversity analysis were filtered with 0.20 µm Supor® membrane filters (PALL life sciences, USA) using a peristaltic pump to concentrate the microbial cells. Total DNA was extracted using Quick DNA Fecal/Soil Microbe MiniPrep DNA Kit (ZYMO RESEARCH, USA) according to the manufacturer’s protocol. The purity of the extracted DNA was assessed on 1.0% agarose gel. Following the Total DNA extraction, the ITS1 (5′TCCGTAGGTGAACCTGCGG′3) and ITS4 (5′ TCCTCCGCTTATTGATATGC 3′) primer pair were used to perform polymerase chain reaction (PCR) in 25 µL reaction mix. The PCR reaction mix contained 12.5ul Taq 2X Master Mix, 9.5 µL nuclease free water, 0.5 µL of both ITS1 and ITS4 primers and 2 µL of the extracted DNA. PCR conditions used include; initial denaturation step at 95 °C for 5 minutes; followed by 32 cycles of denaturation at 95 °C for 30 s; annealing at 55 °C for 1 minute; final extension at 72 °C for 1 minute and final extension at 72 °C for 10 minutes. PCR products were then checked and purified. All PCR products were sequenced using the Illumina Mi-Seq platform at Inqaba Biotechnology (Pretoria, South Africa). Mothur (V 1.39.5) pipeline was used to Analyse the raw sequence data following previous methods^74,75^. Briefly, reads with more than 1% ambiguities or 8% of homopolymers were excluded from processing after merging the paired-end reads into *contigs*. UCHIME was used for discarding chimeric sequences from the remaining *contigs* following the *de novo* method^76^. Subsequently, high quality reads were classified and taxonomically assigned into fungal operational units (OTUs) using UNITE reference database at 95% similarity. A phylogenetic tree was constructed using ITS data set obtained from UNITE database employing the neighbor-Joining method for inferring evolutionary history^77^. The percentage of replicate trees in which the associated taxa clustered together in the bootstrap (500 replicates) was shown next to the branches. The Maximum Composite Likelihood method was used to compute the evolutionary distances^78^ and were are in the units of the number of base substitutions per site. The phylogenetic analysis involved 360 nucleotide sequences. Evolutionary analyses was conducted in MEGA 7 after all ambiguous positions were removed for each sequence pair^79^. Phylotype analysis was performed to identify OTUs similar to the known human and plant pathogens by comparing the obtained fungal phylotypes (OTUs) compared against consensus sequence using BLASTn (≥97% similarity). Fungal Species with the top similarity with OTUs were reported. All the ITS raw datasets were deposited at the NCBI database (https://www.ncbi.nlm.nih.gov/) sequence archive (SRA) with submission No. SUB5214094.

### Statistical analysis

PAST V 3.20 (University of Oslo, USA) statistical software was used to calculate community richness and diversity indices such as chao1 and Simpson-H and plot Canonical correspondence analysis (CCA). XLSTAT (Addinsoft, USA) was used to generate a hierarchically clustered heat-map using the omics tool. Origin pro 8.5 was used to perform one way-ANOVA and Turkey’s significance difference test on the physicochemical properties of the wastewater samples. The online version of Circos software was used to construct Circos graphs for fungal community compositions at the phyla level.

Co-occurrence network analysis was done using Gephi visualization and manipulation (V 0.9.2) combined with the Fruchterman Reingold graph layout algorithm based on spearman’s rank analysis at genus level. Fungal genera with less than 0.05% relative abundance in each wastewater sample were excluded to reduce the rare OTUs. Prior to the analysis, Spearman’s correlation and statistical significance (p < 0.05) were calculated using PAST V 3.20 software. Co-occurrence was valid when Spearman’s correlation coefficient is greater than 0.6 and statistically significant at 0.05 level. Number of edges and nodes, network diameter, modularity, clustering coefficient, average degree, and average path length were calculated to (Gephi V 0.9.2) describe the structural features of the network^4^. The most densely connected node in each module were defined as the “hub” taxa, according to their betweenness centrality, closeness centrality and degree^24^.

## Supplementary information

Supplementary material

## Data Availability

All of the data analysis results obtained during this study are included in this manuscript (and its Supplementary Information files). Raw data for relative abundance of the fungal communities at the different taxonomic levels are available from the corresponding author on reasonable request. All the ITS raw datasets were deposited at the NCBI database (https://www.ncbi.nlm.nih.gov/) sequence archive (SRA) with submission No. SUB5214094.
