# Supplementary material for: Diversity, Co-occurrence and Implications of Fungal Communities in Wastewater Treatment Plants
Source: Sci Rep. 2019 Oct 1;9:14056. doi: 10.1038/s41598-019-50624-z (PMC6773715; doi:10.1038/s41598-019-50624-z)
Supplement: Supplementary file 1 — Supplementary material [file 41598_2019_50624_MOESM1_ESM.docx]

**Diversity, co-occurrence and implications of fungal communities in wastewater treatment plants**

Hailemariam Abrha Assress^1^, Ramganesh Selvarajan^2^, Hlengilizwe Nyoni^1^, Khayalethu Ntushelo^2^, Bhekie B. Mamba^1^ and Titus A.M. Msagati^1^

*^1^University of South Africa, College of Science Engineering and Technology,*

*Nanotechnology and Water Sustainability Research Unit, UNISA Science Campus,*

*P.O. Box 392 UNISA 0003, Florida, 1709 Johannesburg, South Africa*

^2^*University of South Africa, College of Agriculture and Environmental sciences, UNISA Science Campus, P.O. Box 392 UNISA 0003, Florida, 1709 Johannesburg, South Africa*

***Email for correspondence:** [**msagatam@unisa.ac.za**](mailto:msagatam@unisa.ac.za)

**Table S1: Measured metals' level (µg L^-1^) in the investigated wastewater samples (mean ± s.d., *n =3*)**

| **Measured Metal** | **Wastewater samples** | | | | | | | **p-value* (Influent, effluent)** |
| --- | --- | --- | --- | --- | --- | --- | --- | --- |
|  | **DI** | **DE** | **FI** | **FE** | **PI** | **PE** | **HE** |  |
| Calcium (Ca) | 19541.05 ± 1011.84 | 18403.08 ± 275.99 | 11027.19 ± 182.19 | 14639.54 ± 446.10 | 52906.12 ± 2783.75 | 22511.61 ± 331.68 | 15198.63 ± 853.72 | (<0.00001, <0.00001) |
| Cobalt (Co) | 1.31 ± 0.35 | 0.90 ± 0.18 | 1.97 ± 0.22 | 1.15 ± 0.26 | 1.54 ± 0.32 | 1.01 ± 0.19 | 0.86 ± 0.20 | (0.0098, 0.524) |
| Copper (Cu) | 5.08 ± 0.39 | 4.83 ± 0.38 | 6.00 ± 1.03 | 8.47 ± 0.88 | 23.74 ± 9.46 | 11.32 ± 1.26 | 26.09 ± 3.56 | (0.000024, 0.00039) |
| Iron (Fe) | 611.24 ± 12.16 | 339.45 ± 9.85 | 690.98 ± 7.83 | 258.22 ± 10.72 | 272.17 ± 9.65 | 442.28 ± 37.65 | 243.09 ± 18.92 | (0.000013, 0.00052) |
| Magnesium (Mg) | 15141.92 ± 93.93 | 12941.10 ± 20.12 | 5652.41 ± 136.66 | 4717.15 ± 38.07 | 9852.81 ± 71.35 | 6587.39 ± 304.80 | 6931.82 ± 358.15 | (0.000064, <0.00001) |
| Manganese (Mn) | 69.12 ± 0.31 | 2.92 ± 0.54 | 76.50 ± 1.62 | 1.12 ± 0.48 | 311.90 ± 13.06 | 164.53 ± 6.92 | 58.39 ± 7.58 | (<0.00001, <0.00001) |
| Nickel (Ni) | 16.96 ± 1.25 | 5.88 ± 1.60 | 4.29 ± 0.66 | 4.22 ± 1.14 | 30.38 ± 0.81 | 43.43 ± 4.17 | 3.09 ± 0.07 | (<0.00001, 0.0029) |
| Zinc (Zn) | 2.53 ± 0.45 | 5.15 ± 0.69 | 38.16 ± 2.97 | 9.64 ± 0.78 | 107.33 ± 12.05 | 44.15 ± 5.43 | 169.43 ± 10.95 | (<0.00001, 0.000011) |

***(*p<0.05)-* defined as statistically significant**

Table S2: Jaccard’s similarity and distance index

DI DE FI FE PI PE HE

DI 1

DE 0.43007 1

FI 0.4636 0.4078 1

FE 0.34615 0.37602 0.34648 1

PI 0.39927 0.47584 0.42308 0.36182 1

PE 0.44518 0.4377 0.4339 0.41223 0.40066 1

HE 0.39446 0.41216 0.38652 0.38611 0.46442 0.43689 1

Table S3: Relative abundance of the fungal genera in WWTPs and Hospital effluent from Gauteng, South Africa

| ***Genera*** | **DI** | **DE** | **FI** | **FE** | **HE** | **PI** | **PE** |
| --- | --- | --- | --- | --- | --- | --- | --- |
| *Paecilomyces* | 11 | 6 | 1004 | 14 | 1796 | 2238 | 27 |
| *Tricholoma* | 14 | 44 | 24 | 249 | 1294 | 1595 | 49 |
| *Pulchromyces* | 675 | 936 | 229 | 1143 | 1069 | 810 | 400 |
| *Naumovozyma* | 7543 | 431 | 1756 | 2355 | 1059 | 168 | 1066 |
| *Cantharellales_unidentified_1* | 184 | 37 | 54 | 285 | 791 | 190 | 34 |
| *Boletales_unidentified_1* | 680 | 288 | 251 | 160 | 372 | 231 | 223 |
| *Ophiocordyceps* | 194 | 3892 | 224 | 48 | 372 | 858 | 112 |
| *Sebacinales_Group_B_unidentified* | 7 | 22 | 12 | 8 | 314 | 112 | 28 |
| *Thelephoraceae_unidentified* | 7 | 968 | 34 | 32 | 300 | 202 | 24 |
| *Tulasnellaceae_unidentified* | 9 | 6 | 399 | 61 | 255 | 494 | 25 |
| *Inocybe* | 20 | 242 | 97 | 52 | 162 | 50 | 109 |
| *Cyllamyces* | 0 | 0 | 0 | 0 | 155 | 0 | 0 |
| *Piromyces* | 19 | 1 | 6 | 2 | 143 | 0 | 0 |
| *Derxomyces* | 672 | 1594 | 1078 | 1163 | 139 | 390 | 779 |
| *Boletaceae_unidentified* | 0 | 25 | 178 | 28 | 138 | 179 | 89 |
| *Dothideomycetes_unidentified_1* | 68 | 4 | 24 | 6 | 123 | 26 | 90 |
| *Chytridiomycota_unidentified_1_1* | 124 | 29 | 46 | 108 | 119 | 64 | 45 |
| *Mortierella* | 549 | 5 | 214 | 9 | 114 | 83 | 64 |
| *Tremellales_unidentified_1* | 234 | 358 | 193 | 340 | 109 | 152 | 1288 |
| *Hymenochaetales_unidentified_1* | 31 | 31 | 37 | 125 | 103 | 41 | 170 |
| *Massaria* | 29 | 716 | 257 | 21 | 102 | 221 | 28 |
| *Rhodotorula* | 135 | 317 | 119 | 251 | 99 | 105 | 580 |
| *Entoloma* | 1 | 5 | 1 | 24 | 92 | 0 | 7 |
| *Scutellospora* | 31 | 15 | 19 | 12 | 92 | 1 | 6 |
| *Inocybaceae_unidentified* | 0 | 339 | 3 | 22 | 90 | 19 | 13 |
| *Cunninghamella* | 3 | 2 | 0 | 8 | 89 | 1 | 4 |
| *Agaricomycetes_unidentified_1* | 4 | 11 | 4 | 17 | 76 | 19 | 21 |
| *Phymatotrichopsis* | 45 | 35 | 43 | 50 | 73 | 89 | 23 |
| *Albertiniella* | 18 | 19 | 44 | 354 | 67 | 86 | 50 |
| *Glomeraceae_unidentified* | 254 | 29 | 373 | 104 | 58 | 5 | 144 |
| *Pyronemataceae_unidentified* | 128 | 4 | 13 | 12 | 58 | 302 | 7 |
| *Acaulospora* | 13 | 35 | 58 | 21 | 56 | 6 | 16 |
| *Cortinarius* | 6 | 835 | 8 | 9 | 54 | 42 | 69 |
| *Saccharomyces* | 1 | 92 | 3 | 3 | 52 | 8 | 1 |
| *Fungi_unidentified_1_1* | 25 | 23 | 21 | 171 | 47 | 8 | 105 |
| *Peziza* | 1 | 149 | 2 | 6 | 45 | 75 | 3 |
| *Bensingtonia* | 88 | 1 | 117 | 5 | 43 | 415 | 13 |
| *Hydnotrya* | 0 | 0 | 1 | 2 | 41 | 118 | 0 |
| *Ascomycota_unidentified_1_1* | 1 | 20 | 1 | 167 | 38 | 4 | 42 |
| *Glomus* | 4 | 4 | 6 | 2 | 35 | 2 | 36 |
| *Sebacina* | 261 | 15 | 39 | 4 | 33 | 44 | 144 |
| *Astraeus* | 0 | 2 | 1 | 22 | 30 | 14 | 67 |
| *Hypocreaceae_unidentified* | 0 | 0 | 0 | 105 | 26 | 0 | 24 |
| *Glomeromycota_unidentified_1_1* | 35 | 1 | 0 | 15 | 26 | 16 | 2 |
| *Cystoderma* | 1 | 8 | 1 | 9 | 25 | 5 | 36 |
| *Rhodoscypha* | 11 | 2 | 0 | 0 | 25 | 4 | 0 |
| *Septoglomus* | 4 | 4 | 11 | 28 | 24 | 3 | 27 |
| *Sebacinales_unidentified_1* | 105 | 11 | 25 | 13 | 24 | 26 | 134 |
| *Agaricales_unidentified_1* | 6 | 109 | 66 | 30 | 23 | 21 | 132 |
| *Russulaceae_unidentified* | 23 | 71 | 25 | 8 | 23 | 29 | 108 |
| *Cantharellus* | 5 | 29 | 192 | 5 | 22 | 102 | 7 |
| *Helotiales_unidentified_1* | 3 | 11 | 1 | 12 | 21 | 6 | 2 |
| *Paratritirachium* | 0 | 0 | 0 | 0 | 20 | 0 | 0 |
| *Diversisporaceae_unidentified* | 6 | 9 | 13 | 22 | 16 | 27 | 28 |
| *Pseudomassaria* | 8 | 16 | 5 | 19 | 16 | 2 | 2 |
| *Pezizaceae_unidentified* | 3 | 209 | 4 | 13 | 16 | 23 | 6 |
| *Xerocomus* | 0 | 2 | 1 | 6 | 16 | 11 | 51 |
| *Tomentellopsis* | 19 | 2 | 19 | 5 | 16 | 68 | 29 |
| *Gibberella* | 42 | 44 | 3 | 83 | 15 | 4 | 3 |
| *Melanopsamma* | 0 | 0 | 0 | 45 | 15 | 0 | 0 |
| *Coltricia* | 3 | 289 | 4 | 20 | 14 | 85 | 65 |
| *Schizosaccharomyces* | 3 | 4 | 0 | 14 | 13 | 2 | 4 |
| *Puccinia* | 23 | 99 | 12 | 13 | 13 | 13 | 14 |
| *Parasitella* | 0 | 0 | 0 | 13 | 13 | 25 | 42 |
| *Basidiomycota_unidentified_1_1* | 4 | 5 | 4 | 4 | 13 | 3 | 8 |
| *Minimedusa* | 22 | 6466 | 11 | 46 | 12 | 2400 | 63 |
| *Sebacinaceae_unidentified* | 9 | 10 | 2 | 8 | 12 | 4 | 34 |
| *Lindgomyces* | 0 | 3 | 0 | 2 | 12 | 4 | 49 |
| *Gloniaceae_unidentified* | 0 | 0 | 0 | 1 | 12 | 38 | 0 |
| *Hygrocybe* | 5 | 7 | 6 | 66 | 10 | 2 | 108 |
| *Russula* | 11 | 53 | 2847 | 19 | 9 | 21 | 10 |
| *Elaphocordyceps* | 0 | 34 | 3 | 24 | 8 | 9 | 5 |
| *Clavulinaceae_unidentified* | 2 | 58 | 2 | 5 | 8 | 26 | 7 |
| *Wolfiporia* | 1 | 7 | 802 | 4 | 8 | 11 | 152 |
| *Mucorales_unidentified_1* | 1 | 1 | 0 | 3 | 8 | 1 | 129 |
| *Amanita* | 1 | 1 | 0 | 0 | 8 | 35 | 4 |
| *Piedraia* | 0 | 0 | 0 | 0 | 8 | 56 | 0 |
| *Glomeromycetes_unidentified_1* | 22 | 13 | 6 | 239 | 7 | 4 | 3 |
| *Otidea* | 1 | 13 | 0 | 114 | 7 | 10 | 1 |
| *Olpidium* | 8432 | 24 | 1374 | 8 | 7 | 8 | 75 |
| *Genea* | 38 | 76 | 6 | 1 | 7 | 14 | 310 |
| *Nectriaceae_unidentified* | 1 | 2 | 0 | 0 | 7 | 0 | 1 |
| *Reddellomyces* | 1 | 0 | 0 | 0 | 7 | 0 | 1 |
| *Knufia* | 28 | 8 | 7 | 91 | 6 | 12 | 24 |
| *Funneliformis* | 112 | 274 | 15 | 26 | 6 | 2 | 5 |
| *Lecanicillium* | 0 | 0 | 0 | 1 | 6 | 8 | 0 |
| *Scutellinia* | 0 | 1 | 1 | 0 | 6 | 0 | 3 |
| *Trichoderma* | 0 | 0 | 0 | 23 | 5 | 0 | 4 |
| *Lasiosphaeriaceae_unidentified* | 62 | 2 | 169 | 14 | 5 | 3 | 37 |
| *Falcocladium* | 1 | 6 | 0 | 11 | 5 | 1 | 5 |
| *Mucor* | 0 | 3 | 1 | 9 | 5 | 0 | 3 |
| *Atheliaceae_unidentified* | 0 | 11 | 6 | 8 | 5 | 24 | 51 |
| *Sordariomycetes_unidentified_1* | 90 | 2 | 9 | 1 | 5 | 0 | 11 |
| *Microbotryales_unidentified_1* | 0 | 0 | 0 | 1 | 5 | 2 | 0 |
| *Pichia* | 2 | 0 | 1 | 0 | 5 | 0 | 3 |
| *Synchytrium* | 19 | 6 | 24 | 14 | 4 | 2 | 16 |
| *Pseudotomentella* | 3 | 9551 | 58 | 12 | 4 | 3137 | 20 |
| *Rhizophydium* | 0 | 0 | 1 | 9 | 4 | 0 | 0 |
| *Xylaria* | 0 | 0 | 1 | 2 | 4 | 2 | 0 |
| *Sporothrix* | 0 | 0 | 0 | 0 | 4 | 0 | 5 |
| *Pannaria* | 5 | 20 | 8 | 52 | 3 | 4 | 24 |
| *Hypocrea* | 0 | 0 | 0 | 41 | 3 | 0 | 4 |
| *Clavulina* | 61 | 1689 | 22 | 8 | 3 | 16 | 1469 |
| *Sporobolomyces* | 0 | 2 | 1 | 8 | 3 | 0 | 1 |
| *Cortinariaceae_unidentified* | 2 | 3 | 0 | 5 | 3 | 8 | 79 |
| *Candida* | 355 | 56 | 3 | 4 | 3 | 18 | 10 |
| *Antherospora* | 0 | 0 | 4 | 0 | 3 | 0 | 2 |
| *Mycenaceae_unidentified* | 0 | 0 | 0 | 0 | 3 | 0 | 0 |
| *Eucasphaeria* | 0 | 1 | 4 | 181 | 2 | 3 | 5 |
| *Ochroconis* | 4 | 7 | 0 | 55 | 2 | 4 | 190 |
| *Tomentella* | 0 | 44 | 3 | 31 | 2 | 2 | 8 |
| *Saccharomycetaceae_unidentified* | 0 | 10 | 0 | 29 | 2 | 6 | 448 |
| *Lactarius* | 3 | 2 | 10 | 5 | 2 | 8 | 10 |
| *Odontia* | 14 | 0 | 2 | 4 | 2 | 1 | 2 |
| *Gymnopus* | 1 | 0 | 0 | 4 | 2 | 5 | 0 |
| *Thelephorales_unidentified_1* | 0 | 0 | 0 | 3 | 2 | 0 | 0 |
| *Caloplaca* | 23 | 25 | 7 | 2 | 2 | 30 | 5 |
| *Glaciozyma* | 0 | 0 | 1 | 2 | 2 | 2 | 7 |
| *Coemansia* | 1 | 1 | 28 | 1 | 2 | 0 | 4 |
| *Atractiellales_unidentified_1* | 0 | 216 | 5 | 1 | 2 | 10 | 36 |
| *Hydnobolites* | 0 | 29 | 0 | 1 | 2 | 7 | 0 |
| *Sympoventuria* | 1 | 38 | 1 | 0 | 2 | 3 | 0 |
| *Tothia* | 0 | 5 | 144 | 0 | 2 | 0 | 3 |
| *Phylloporus* | 0 | 0 | 0 | 0 | 2 | 0 | 10 |
| *Tricholosporum* | 0 | 0 | 0 | 0 | 2 | 0 | 0 |
| *Pleosporaceae_unidentified* | 0 | 0 | 0 | 0 | 2 | 1 | 1 |
| *Pilobolus* | 0 | 0 | 0 | 0 | 2 | 0 | 0 |
| *Cephalotheca* | 0 | 0 | 0 | 0 | 2 | 1 | 0 |
| *Trichosporon* | 0 | 0 | 0 | 0 | 2 | 0 | 1 |
| *Acaulosporaceae_unidentified* | 4 | 5 | 0 | 44 | 1 | 0 | 2 |
| *Junghuhnia* | 4 | 2 | 1 | 41 | 1 | 2 | 1 |
| *Penicillium* | 663 | 2 | 61 | 32 | 1 | 2 | 2 |
| *Morchella* | 126 | 9 | 15 | 7 | 1 | 4 | 4 |
| *Capronia* | 1 | 29 | 1 | 5 | 1 | 1 | 2 |
| *Orpinomyces* | 0 | 1 | 0 | 5 | 1 | 0 | 0 |
| *Macalpinomyces* | 2 | 0 | 2 | 3 | 1 | 0 | 17 |
| *Boletellus* | 1 | 2 | 2 | 3 | 1 | 3 | 1 |
| *Amaurodon* | 2 | 5 | 1 | 2 | 1 | 4 | 1 |
| *Limacella* | 0 | 0 | 0 | 2 | 1 | 0 | 0 |
| *Cunninghamella* | 1 | 2 | 1 | 1 | 1 | 0 | 3 |
| *Entolomataceae_unidentified* | 1 | 0 | 1 | 1 | 1 | 1 | 1 |
| *Ramicandelaber* | 0 | 36 | 0 | 1 | 1 | 31 | 3 |
| *Galerina* | 0 | 0 | 0 | 1 | 1 | 0 | 0 |
| *Tetrapisispora* | 0 | 0 | 0 | 1 | 1 | 0 | 0 |
| *Udeniomyces* | 4 | 1 | 3 | 0 | 1 | 0 | 1 |
| *Geoglossum* | 2 | 0 | 1 | 0 | 1 | 0 | 0 |
| *Marasmius* | 1 | 0 | 1 | 0 | 1 | 0 | 0 |
| *Priceomyces* | 1 | 0 | 0 | 0 | 1 | 0 | 0 |
| *Cryphonectria* | 1 | 0 | 0 | 0 | 1 | 0 | 35 |
| *Helicostylum* | 0 | 1 | 0 | 0 | 1 | 0 | 0 |
| *Polyscytalum* | 0 | 1 | 0 | 0 | 1 | 0 | 0 |
| *Rhizophydiales_unidentified_1* | 0 | 0 | 1 | 0 | 1 | 0 | 0 |
| *Canalisporium* | 0 | 0 | 1 | 0 | 1 | 0 | 0 |
| *Clavaria* | 0 | 0 | 0 | 0 | 1 | 0 | 0 |
| *Craterellus* | 0 | 0 | 0 | 0 | 1 | 0 | 3 |
| *Sarcodon* | 0 | 0 | 0 | 0 | 1 | 0 | 0 |
| *Trechispora* | 0 | 0 | 0 | 0 | 1 | 0 | 0 |
| *Tulasnella* | 0 | 0 | 0 | 0 | 1 | 0 | 0 |
| *Alphamyces* | 0 | 0 | 0 | 0 | 1 | 1 | 0 |
| *Hortaea* | 0 | 0 | 0 | 0 | 1 | 0 | 0 |
| *Stagonospora* | 0 | 0 | 0 | 0 | 1 | 0 | 0 |
| *Cyphellophora* | 0 | 0 | 0 | 0 | 1 | 0 | 0 |
| *Ambispora* | 0 | 0 | 0 | 0 | 1 | 0 | 0 |
| *Incertae_sedis_3_unidentified* | 0 | 0 | 0 | 0 | 1 | 0 | 0 |
| *Ophiostoma* | 0 | 0 | 0 | 0 | 1 | 0 | 1 |
| *Phialosimplex* | 0 | 0 | 1 | 2512 | 0 | 0 | 0 |
| *Aspergillus* | 8 | 1 | 0 | 2349 | 0 | 2 | 2 |
| *Venturiaceae_unidentified* | 1 | 14 | 17 | 82 | 0 | 1 | 7 |
| *Purpureocillium* | 0 | 0 | 0 | 72 | 0 | 0 | 0 |
| *Fennellia* | 0 | 1 | 0 | 71 | 0 | 0 | 0 |
| *Pilaira* | 1 | 0 | 0 | 58 | 0 | 0 | 0 |
| *Neophaeosphaeria* | 0 | 0 | 0 | 44 | 0 | 0 | 0 |
| *Cladophialophora* | 1 | 33 | 2 | 32 | 0 | 1 | 26 |
| *Mycocladus* | 0 | 0 | 0 | 17 | 0 | 0 | 0 |
| *Trichocomaceae_unidentified* | 0 | 0 | 0 | 16 | 0 | 0 | 0 |
| *Clavicipitaceae_unidentified* | 0 | 0 | 0 | 14 | 0 | 0 | 0 |
| *Emmonsia* | 2 | 0 | 1 | 13 | 0 | 0 | 1 |
| *Ophiocordycipitaceae_unidentified* | 0 | 0 | 0 | 10 | 0 | 0 | 0 |
| *Endothiella* | 0 | 0 | 1 | 8 | 0 | 0 | 49 |
| *Capnobotryella* | 0 | 0 | 0 | 8 | 0 | 0 | 0 |
| *Cronartium* | 1 | 0 | 0 | 7 | 0 | 0 | 0 |
| *Chaetothyriales_unidentified_1* | 9 | 126 | 10 | 5 | 0 | 51 | 11 |
| *Davidiella* | 0 | 0 | 0 | 5 | 0 | 0 | 0 |
| *Chrysosporium* | 0 | 0 | 0 | 5 | 0 | 0 | 0 |
| *Rhizomucor* | 0 | 0 | 0 | 5 | 0 | 0 | 0 |
| *Sticta* | 0 | 0 | 0 | 5 | 0 | 0 | 0 |
| *Sordariales_unidentified_1* | 0 | 0 | 0 | 5 | 0 | 0 | 0 |
| *Pleochaeta* | 0 | 1 | 0 | 4 | 0 | 0 | 1 |
| *Phaeocollybia* | 0 | 0 | 0 | 4 | 0 | 0 | 0 |
| *Eurotiales_unidentified_1* | 0 | 0 | 0 | 4 | 0 | 0 | 1 |
| *Tuber* | 2 | 6 | 0 | 3 | 0 | 0 | 15 |
| *Kazachstania* | 2 | 0 | 0 | 3 | 0 | 0 | 0 |
| *Mycena* | 1 | 6 | 0 | 3 | 0 | 5 | 0 |
| *Arthrobotrys* | 0 | 0 | 1 | 3 | 0 | 0 | 0 |
| *Verticillium* | 0 | 0 | 0 | 3 | 0 | 0 | 0 |
| *Hyphodiscus* | 13 | 3 | 1 | 2 | 0 | 1 | 0 |
| *Baeomyces* | 4 | 0 | 3 | 2 | 0 | 0 | 1 |
| *Elaphomycetaceae_unidentified* | 4 | 0 | 1 | 2 | 0 | 0 | 1 |
| *Phyllosticta* | 1 | 14 | 14 | 2 | 0 | 3 | 2 |
| *Lachancea* | 1 | 4 | 0 | 2 | 0 | 0 | 0 |
| *Erysiphe* | 1 | 0 | 0 | 2 | 0 | 0 | 0 |
| *Stachybotrys* | 0 | 2 | 2 | 2 | 0 | 3 | 34 |
| *Talaromyces* | 0 | 1 | 0 | 2 | 0 | 0 | 0 |
| *Rickenella* | 0 | 0 | 0 | 2 | 0 | 0 | 0 |
| *Cladosporium* | 0 | 0 | 0 | 2 | 0 | 0 | 0 |
| *Protoblastenia* | 0 | 0 | 0 | 2 | 0 | 0 | 0 |
| *Barnettozyma* | 0 | 0 | 0 | 2 | 0 | 0 | 0 |
| *Glomerellaceae_unidentified* | 0 | 0 | 0 | 2 | 0 | 0 | 3 |
| *Hysterangiales_unidentified_1* | 13 | 1 | 4 | 1 | 0 | 1 | 4 |
| *Auriculariales_unidentified_1* | 10 | 1 | 2 | 1 | 0 | 0 | 9 |
| *Fusicladium* | 2 | 2 | 1 | 1 | 0 | 0 | 1 |
| *Monoblepharidales_unidentified_1* | 1 | 0 | 0 | 1 | 0 | 0 | 2 |
| *Boletus* | 0 | 12 | 0 | 1 | 0 | 5 | 0 |
| *Archaeosporaceae_unidentified* | 0 | 3 | 1 | 1 | 0 | 0 | 17 |
| *Peltigera* | 0 | 2 | 0 | 1 | 0 | 4 | 0 |
| *Kondoa* | 0 | 1 | 0 | 1 | 0 | 0 | 0 |
| *Pirella* | 0 | 1 | 0 | 1 | 0 | 0 | 0 |
| *Malasseziales_unidentified_1* | 0 | 1 | 0 | 1 | 0 | 0 | 0 |
| *Chaetosphaeriales_unidentified_1* | 0 | 0 | 8 | 1 | 0 | 0 | 0 |
| *Fomitiporia* | 0 | 0 | 1 | 1 | 0 | 0 | 2 |
| *Mortierellaceae_unidentified* | 0 | 0 | 1 | 1 | 0 | 0 | 5 |
| *Hypomyces* | 0 | 0 | 1 | 1 | 0 | 1 | 5 |
| *Clavariadelphus* | 0 | 0 | 0 | 1 | 0 | 0 | 0 |
| *Ganoderma* | 0 | 0 | 0 | 1 | 0 | 0 | 0 |
| *Gerronema* | 0 | 0 | 0 | 1 | 0 | 0 | 6 |
| *Hydnellum* | 0 | 0 | 0 | 1 | 0 | 0 | 0 |
| *Phaeolepiota* | 0 | 0 | 0 | 1 | 0 | 0 | 0 |
| *Pleurotus* | 0 | 0 | 0 | 1 | 0 | 0 | 0 |
| *Resinicium* | 0 | 0 | 0 | 1 | 0 | 0 | 0 |
| *Pateramyces* | 0 | 0 | 0 | 1 | 0 | 0 | 0 |
| *Lewia* | 0 | 0 | 0 | 1 | 0 | 0 | 0 |
| *Microsporum* | 0 | 0 | 0 | 1 | 0 | 0 | 0 |
| *Verruculopsis* | 0 | 0 | 0 | 1 | 0 | 0 | 0 |
| *Entyloma* | 0 | 0 | 0 | 1 | 0 | 0 | 1 |
| *Tilletiopsis* | 0 | 0 | 0 | 1 | 0 | 0 | 0 |
| *Pacispora* | 0 | 0 | 0 | 1 | 0 | 0 | 0 |
| *Backusella* | 0 | 0 | 0 | 1 | 0 | 0 | 1 |
| *Malassezia* | 0 | 0 | 0 | 1 | 0 | 0 | 0 |
| *Lecanoromycetes_unidentified_1* | 0 | 0 | 0 | 1 | 0 | 0 | 0 |
| *Teloschistaceae_unidentified* | 0 | 0 | 0 | 1 | 0 | 0 | 0 |
| *Cryptosporiopsis* | 0 | 0 | 0 | 1 | 0 | 0 | 0 |
| *Leveillula* | 0 | 0 | 0 | 1 | 0 | 0 | 1 |
| *Pseudoidium* | 0 | 0 | 0 | 1 | 0 | 0 | 0 |
| *Neocallimastix* | 0 | 0 | 0 | 1 | 0 | 0 | 0 |
| *Wilcoxina* | 0 | 0 | 0 | 1 | 0 | 0 | 0 |
| *Botryozyma* | 0 | 0 | 0 | 1 | 0 | 0 | 0 |
| *Metschnikowia* | 0 | 0 | 0 | 1 | 0 | 0 | 0 |
| *Ogataea* | 0 | 0 | 0 | 1 | 0 | 0 | 1 |
| *Gaeumannomyces* | 0 | 0 | 0 | 1 | 0 | 0 | 0 |
| *Gondwanamyces* | 0 | 0 | 0 | 1 | 0 | 0 | 0 |
| *Hirsutella* | 0 | 0 | 0 | 1 | 0 | 0 | 0 |
| *Nalanthamala* | 0 | 0 | 0 | 1 | 0 | 0 | 0 |
| *Phomopsis* | 0 | 0 | 0 | 1 | 0 | 0 | 0 |
| *Syzygospora* | 0 | 0 | 0 | 1 | 0 | 0 | 0 |
| *Urocystis* | 0 | 0 | 0 | 1 | 0 | 0 | 0 |
| *Ustilago* | 0 | 0 | 0 | 1 | 0 | 0 | 0 |
| *Umbelopsis* | 327 | 0 | 9 | 0 | 0 | 1 | 4 |
| *Fonsecaea* | 161 | 0 | 4 | 0 | 0 | 0 | 0 |
| *Corollospora* | 20 | 0 | 3 | 0 | 0 | 0 | 0 |
| *Cryptodiscus* | 7 | 0 | 2 | 0 | 0 | 0 | 0 |
| *Aspicilia* | 6 | 0 | 1 | 0 | 0 | 0 | 0 |
| *Kurtzmanomyces* | 4 | 4 | 14 | 0 | 0 | 0 | 0 |
| *Erysiphaceae_unidentified* | 4 | 0 | 0 | 0 | 0 | 0 | 0 |
| *Melanconiella* | 3 | 32 | 6 | 0 | 0 | 12 | 23 |
| *Pleosporales_unidentified_1* | 3 | 2 | 0 | 0 | 0 | 1 | 5 |
| *Periconia* | 3 | 0 | 2 | 0 | 0 | 0 | 0 |
| *Pyrigemmula* | 2 | 1 | 0 | 0 | 0 | 3 | 0 |
| *Cistella* | 2 | 0 | 2 | 0 | 0 | 0 | 0 |
| *Zygosaccharomyces* | 2 | 0 | 1 | 0 | 0 | 1 | 0 |
| *Hymenochaete* | 2 | 0 | 0 | 0 | 0 | 0 | 1 |
| *Ramaria* | 2 | 0 | 0 | 0 | 0 | 0 | 0 |
| *Albatrellaceae_unidentified* | 2 |  | 0 | 0 | 0 | 0 | 0 |
| *Pichiaceae_unidentified* | 1 | 138 | 0 | 0 | 0 | 0 | 0 |
| *Pezizales_unidentified_1* | 1 | 5 | 0 | 0 | 0 | 0 | 0 |
| *Ceratobasidium* | 1 | 1 | 0 | 0 | 0 | 0 | 2 |
| *Rhizophlyctis* | 1 | 1 | 0 | 0 | 0 | 0 | 0 |
| *Bionectria* | 1 | 0 | 1 | 0 | 0 | 0 | 1 |
| *Hygrophoraceae_unidentified* | 1 | 0 | 0 | 0 | 0 | 0 | 0 |
| *Cladoriella* | 1 | 0 | 0 | 0 | 0 | 0 | 0 |
| *Phoma* | 1 | 0 | 0 | 0 | 0 | 0 | 0 |
| *Elaphomyces* | 1 | 0 | 0 | 0 | 0 | 0 | 1 |
| *Eurotiomycetes_unidentified_1* | 1 | 0 | 0 | 0 | 0 | 2 | 0 |
| *Herpotrichiellaceae_unidentified* | 1 | 0 | 0 | 0 | 0 | 0 | 0 |
| *Verrucaria* | 1 | 0 | 0 | 0 | 0 | 0 | 1 |
| *Bryoria* | 1 | 0 | 0 | 0 | 0 | 0 | 0 |
| *Glarea* | 1 | 0 | 0 | 0 | 0 | 0 | 0 |
| *Ustilentyloma* | 1 | 0 | 0 | 0 | 0 | 0 | 0 |
| *Pucciniomycetes_unidentified_1* | 1 | 0 | 0 | 0 | 0 | 0 | 0 |
| *Fusarium* | 1 | 0 | 0 | 0 | 0 | 0 | 1 |
| *Savoryella* | 1 | 0 | 0 | 0 | 0 | 0 | 0 |
| *Tolypocladium* | 1 | 0 | 0 | 0 | 0 | 0 | 0 |
| *Archaeorhizomycetes_unidentified_1* | 0 | 15 | 0 | 0 | 0 | 0 | 0 |
| *Circinella* | 0 | 13 | 0 | 0 | 0 | 1 | 0 |
| *Helvella* | 0 | 7 | 2 | 0 | 0 | 0 | 3 |
| *Coprinopsis* | 0 | 7 | 1 | 0 | 0 | 4 | 0 |
| *Lacazia* | 0 | 4 | 0 | 0 | 0 | 0 | 0 |
| *Guignardia* | 0 | 3 | 0 | 0 | 0 | 0 | 0 |
| *Leptosphaeriaceae_unidentified* | 0 | 3 | 0 | 0 | 0 | 0 | 2 |
| *Leotiomycetes_unidentified_1* | 0 | 3 | 0 | 0 | 0 | 0 | 0 |
| *Geosmithia* | 0 | 2 | 2 | 0 | 0 | 0 | 0 |
| *Dirinaria* | 0 | 2 | 1 | 0 | 0 | 0 | 0 |
| *Gymnopilus* | 0 | 2 | 0 | 0 | 0 | 0 | 0 |
| *Oidiodendron* | 0 | 2 | 0 | 0 | 0 | 0 | 0 |
| *Zychaea* | 0 | 2 | 0 | 0 | 0 | 0 | 0 |
| *Kappamyces* | 0 | 1 | 1 | 0 | 0 | 0 | 4 |
| *Phaeococcomyces* | 0 | 1 | 1 | 0 | 0 | 0 | 0 |
| *Hydnangiaceae_unidentified* | 0 | 1 | 0 | 0 | 0 | 0 | 0 |
| *Physalacriaceae_unidentified* | 0 | 1 | 0 | 0 | 0 | 0 | 0 |
| *Suillus* | 0 | 1 | 0 | 0 | 0 | 0 | 0 |
| *Helicoma* | 0 | 1 | 0 | 0 | 0 | 0 | 0 |
| *Meliniomyces* | 0 | 1 | 0 | 0 | 0 | 0 | 0 |
| *Orbiliomycetes_unidentified_1* | 0 | 1 | 0 | 0 | 0 | 0 | 0 |
| *Trichophaea* | 0 | 1 | 0 | 0 | 0 | 0 | 0 |
| *Boliniaceae_unidentified* | 0 | 1 | 0 | 0 | 0 | 0 | 0 |
| *Gliomastix* | 0 | 1 | 0 | 0 | 0 | 0 | 0 |
| *Fellomyces* | 0 | 0 | 3 | 0 | 0 | 0 | 3 |
| *Cora* | 0 | 0 | 1 | 0 | 0 | 0 | 2 |
| *Crucibulum* | 0 | 0 | 1 | 0 | 0 | 0 | 0 |
| *Schizophyllum* | 0 | 0 | 1 | 0 | 0 | 0 | 0 |
| *Botryotinia* | 0 | 0 | 1 | 0 | 0 | 0 | 0 |
| *Claussenomyces* | 0 | 0 | 1 | 0 | 0 | 0 | 0 |
| *Coccomyces* | 0 | 0 | 1 | 0 | 0 | 0 | 0 |
| *Podosphaera* | 0 | 0 | 1 | 0 | 0 | 0 | 0 |
| *Calosphaeria* | 0 | 0 | 1 | 0 | 0 | 0 | 0 |
| *Lasiosphaeris* | 0 | 0 | 1 | 0 | 0 | 0 | 1 |
| *Armirilla* | 0 | 0 | 0 | 0 | 0 | 0 | 1 |
| *Battarrea* | 0 | 0 | 0 | 0 | 0 | 1 | 0 |
| *Camarophyllus* | 0 | 0 | 0 | 0 | 0 | 0 | 1 |
| *Ceratobasidiaceae_unidentified* | 0 | 0 | 0 | 0 | 0 | 0 | 3 |
| *Corticiaceae_unidentified* | 0 | 0 | 0 | 0 | 0 | 1 | 0 |
| *Entomocorticium* | 0 | 0 | 0 | 0 | 0 | 1 | 0 |
| *Peniophoraceae_unidentified* | 0 | 0 | 0 | 0 | 0 | 1 | 0 |
| *Polyporales_unidentified_1* | 0 | 0 | 0 | 0 | 0 | 3 | 0 |
| *Rhodocollybia* | 0 | 0 | 0 | 0 | 0 | 0 | 1 |
| *Archaeorhizomyces* | 0 | 0 | 0 | 0 | 0 | 0 | 1 |
| *Cenococcum* | 0 | 0 | 0 | 0 | 0 | 1 | 0 |
| *Corynespora* | 0 | 0 | 0 | 0 | 0 | 0 | 1 |
| *Mycosphaerella* | 0 | 0 | 0 | 0 | 0 | 0 | 1 |
| *Eurotium* | 0 | 0 | 0 | 0 | 0 | 0 | 1 |
| *Polyblastia* | 0 | 0 | 0 | 0 | 0 | 1 | 0 |
| *Utharomyces* | 0 | 0 | 0 | 0 | 0 | 1 | 0 |
| *Retroconis* | 0 | 0 | 0 | 0 | 0 | 1 | 0 |
| *Cladia* | 0 | 0 | 0 | 0 | 0 | 0 | 1 |
| *Cladonia* | 0 | 0 | 0 | 0 | 0 | 2 | 0 |
| *Diploschistes* | 0 | 0 | 0 | 0 | 0 | 1 | 0 |
| *Lepraria* | 0 | 0 | 0 | 0 | 0 | 0 | 1 |
| *Rhizocarpon* | 0 | 0 | 0 | 0 | 0 | 0 | 5 |
| *Stereocaulon* | 0 | 0 | 0 | 0 | 0 | 0 | 1 |
| *Dermateaceae_unidentified* | 0 | 0 | 0 | 0 | 0 | 0 | 1 |
| *Hyaloscyphaceae_unidentified* | 0 | 0 | 0 | 0 | 0 | 2 | 0 |
| *Pseudoplectania* | 0 | 0 | 0 | 0 | 0 | 1 | 0 |
| *Pucciniastrum* | 0 | 0 | 0 | 0 | 0 | 0 | 2 |
| *Blastobotrys* | 0 | 0 | 0 | 0 | 0 | 0 | 2 |
| *Acremonium* | 0 | 0 | 0 | 0 | 0 | 1 | 0 |
| *Ascotaiwania* | 0 | 0 | 0 | 0 | 0 | 0 | 1 |
| *Ceratocystis* | 0 | 0 | 0 | 0 | 0 | 0 | 1 |
| *Hypocreales_unidentified_1* | 0 | 0 | 0 | 0 | 0 | 0 | 4 |
| *Microascales_unidentified_1* | 0 | 0 | 0 | 0 | 0 | 0 | 1 |
| *Hannaella* | 0 | 0 | 0 | 0 | 0 | 0 | 1 |
| *Sporisorium* | 0 | 0 | 0 | 0 | 0 | 0 | 1 |
| *Absidia* | 0 | 0 | 0 | 0 | 0 | 0 | 2 |


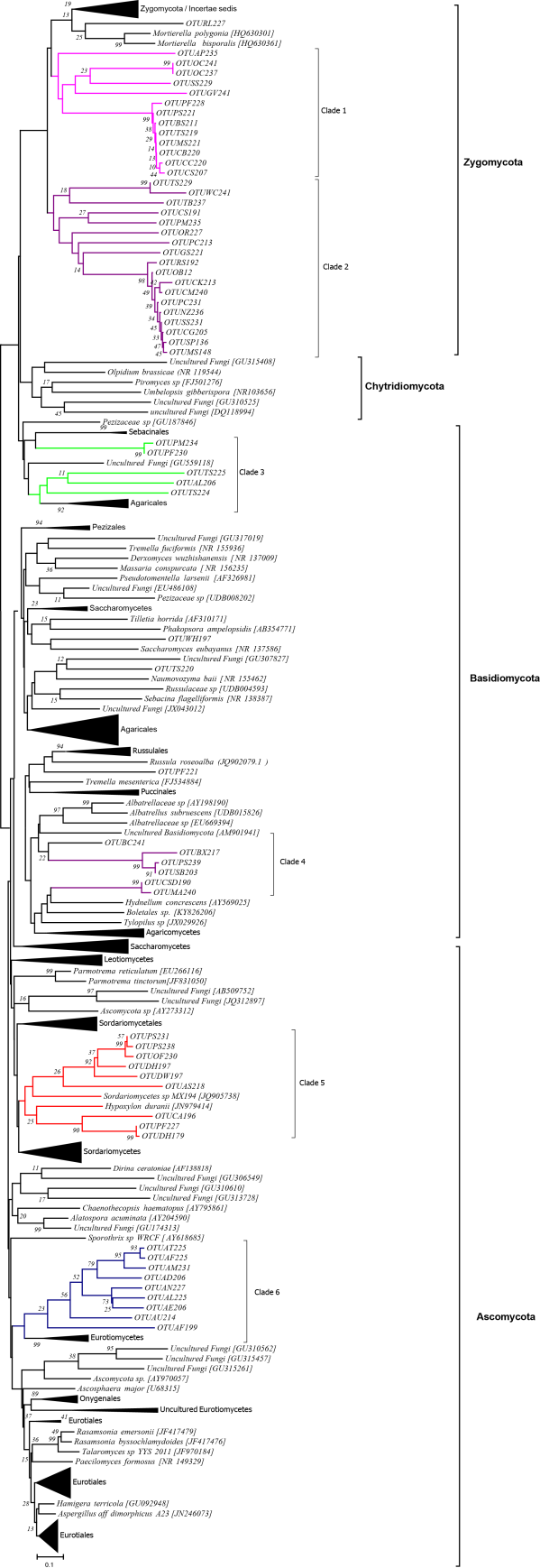


Figure S1: Phylogenetic tree of sequences of fungal OTUs in WWTPs and reference sequences from UNITE database.


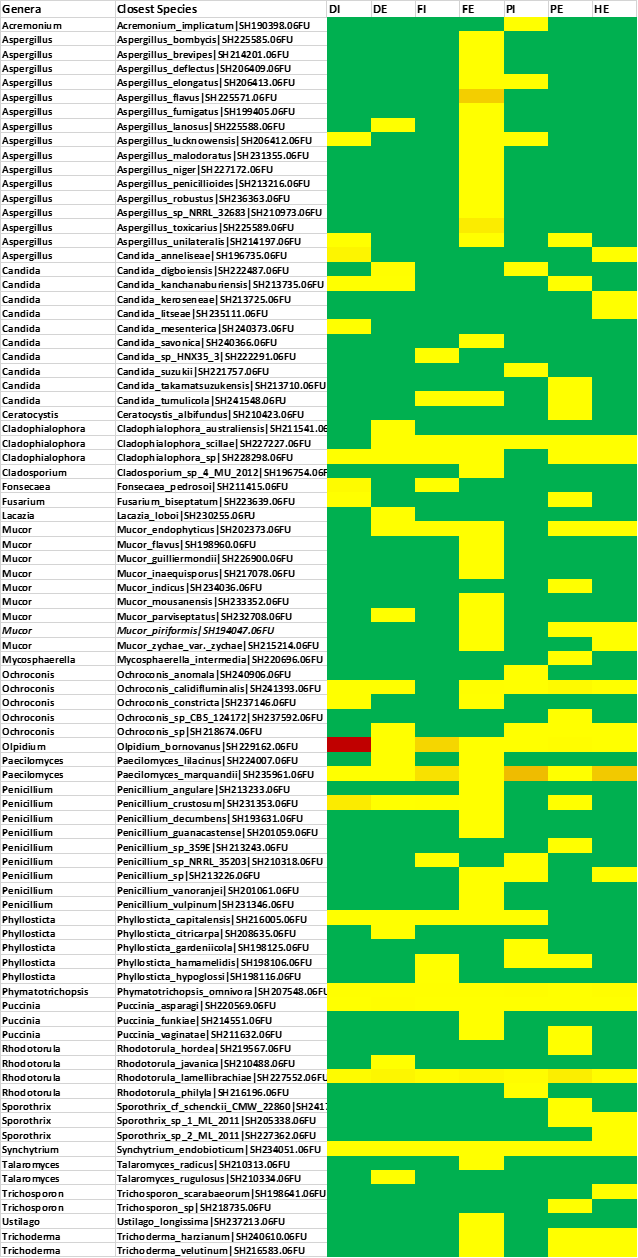


Figure S2: Relative abundance of recovered fungal genera belonging to the reported potential human and/or plant pathogens ( High Medium Low)

| **OTU IDs** | **Genus** | **Closest species similarity in Genebank with accession numbers** | **Similarity (%)** | **Pathogenicity** |
| --- | --- | --- | --- | --- |
| OTU42 | *Aspergillus* | *Aspergillus fumigatus isolate 16 (MH345856)* | 100 | Human and plant |
| OTU22 | *Aspergillus* | *Aspergillus niger*  *strain 102 (MG725819)* | 100 | Human and plant |
| OTU28 | *Cladosporium* | *Cladosporium sphaerospermum*  *isolate AW39N (KP735247)* | 97 | Plant |
| OTU11 | *Aspergillus* | *Aspergillus flavus*  *strain BF25 (MK215722)* | 97 | Human and plant |
| OTU44 | *Olpidium* | *Olpidium bornovanus isolate GT-O1 ( EU937728)* | 98.09 | Plant |
| OTU56 | *Penicillium* | *Penicillium crustosum isolate F36-03 (KX664385)* | 99.22 | Human |
| OTU121 | *Phymatotrichopsis* | *Phymatotrichopsis omnivora strain ATCC 32448 (EF494038)* | 97.54 | Plant |
| OTU100 | *Synchytrium* | *Synchytrium endobioticum voucher DAOM_Dearness3250*  *(KF160872)* | 100 | Plant |
| OTU98 | *Phyllosticta* | *Phyllosticta capitalensis strain FRAH11 ( MK443377)* | 100 | Plant |
| OTU613 | *Mucor* | *Mucor indicus strain ATCC MYA-4678 (HQ263357)* | 98.98 | Human |
| OTU432 | *Acremonium* | *Acremonium implicatum LF30 ( GU951762)* | 99.77 | Human |
| OTU122 | *Ceratocystis* | *Ceratocystis albifundus isolate CMW17773 ( DQ250382)* | 97.16 | Plant |
| OTU330 | *Mycosphaerella* | *Mycosphaerella intermedia strain CMW37290 (KF420416)* | 100 | Plant |
| OTU1123 | *Ustilago* | *Ustilago longissima var. macrospora voucher CBS160.22 (JN367305)* | 99.51 | Plant |
| OTU892 | *Cladophialophora* | *Cladophialophora scillae strain CBS 116461 ( EU035412)* | 97.11 | Human |
| OTU354 | *Fonsecaea* | *Fonsecaea pedrosoi ( MF173064)* | 100 | Human |
| OTU212 | *Lacazia* | *Lacazia loboi strain 35-RNC ( EU167510)* | 99.4 | Human |
| OTU478 | *Ochroconis* | *Ochroconis constricta strain GSV 1 (KF437521)* | 98.5 | Human |
| OTU2460 | *Sporothrix* | *Sporothrix schenckii isolate C3037 ( KJ999885)* | 99.73 | Human |
| OTU252 | *Trichoderma* | *Trichoderma harzianum voucher HMQAU120015 (KP747445)* | 100 | Human |

Table S4: Pathogenic fungal phylotypes identified in the wastewater treatment plants (top scoring hits amongst published sequences in NCBI GenBank)
